# Relationship of electrochemical performance and biofilm development of *Desulfuromonas acetexigens* and *Geobacter sulfurreducens* in microbial electrolysis cells

**DOI:** 10.3389/fmicb.2026.1753230

**Published:** 2026-03-10

**Authors:** Max Rümenapf, Harald Horn, Andrea Hille-Reichel

**Affiliations:** 1 Engler-Bunte-Institut, Water Chemistry and Water Technology, Karlsruhe Institute of Technology (KIT), Karlsruhe, Germany; 2 DVGW Research Centre, Water Chemistry and Water Technology, Karlsruhe, Germany

**Keywords:** biofilm characterization, biofilm morphology, *Desulfuromonas acetexigens*, *Geobacter sulfurreducens*, hydrogen production, single-chamber microbial electrolysis cell

## Abstract

*Desulfuromonas acetexigens* has gained attention as a biocatalyst in microbial electrolysis cells (MECs) due to its inability to utilize hydrogen as an electron donor, which favors beneficial Coulombic efficiencies (CE). In this study, the electrochemical performance and biofilm morphology of *D. acetexigens* were compared with the model organism *Geobacter sulfurreducens* in flow cell MECs. Biofilm morphology was assessed non-invasively via optical coherence tomography (OCT), providing insight into quantitative parameters, including spatially resolved thickness, biovolume and anode surface coverage. While both species achieved similar maximum current densities when cultivated under identical conditions, *D. acetexigens* biofilms established faster, generating current after ~4 days, whereas *G. sulfurreducens* exhibited a lag phase of ~8 days. Limitations of extracellular electron transfer already occurred at lower average biofilm volumes for *D. acetexigens* ($\left({\bar{BV}}_{\bar{J}\max}\right)$ ≈ 16 ± 6 μm^3^ μm^−2^) than for *Geobacter* (${\bar{BV}}_{\bar{J}\max}$ ≈ 40 ± 7 μm^3^ μm^−2^). One monocultural *D. acetexigens* cultivation revealed a CE of ~96%, consistent with no detectable hydrogen utilization under the tested condition, while some cultivations showed net acetate increases. Phylogenetic analyses of the latter indicated niche dominance of the target EAM despite homoacetogenic and clostridial contaminants. Production of short-chain fatty acids suggested interspecies metabolic interaction and led to the hypothesis of an electrode-mediated ethanol to acetate fermentation by electroactive microorganisms and ethanol-utilizing contaminants such as the homoacetogen *Sporomusa sphaeroides*.

## Graphical Abstract

**Figure:**
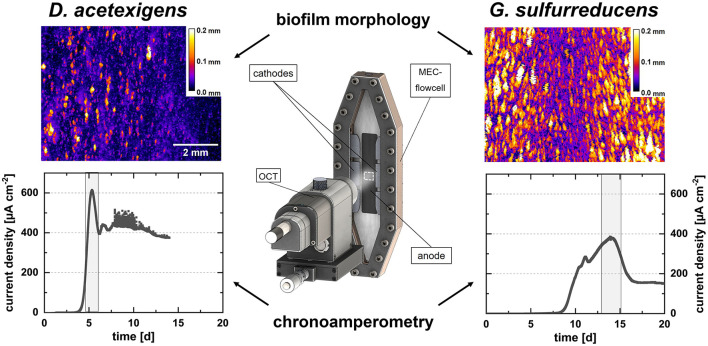

## Highlights

- In *D. acetexigens* cultivations, current density is limited already at lower biofilm volumes (${\bar{BV}}_{\bar{J}\max}\approx$ 16 μm^3^ μm^−2^) compared to *G. sulfurreducens* (${\bar{BV}}_{\bar{J}\max}\approx$ 40 μm^3^ μm^−2^).
- Exponential increase of current generation after inoculation is two times faster in *D. acetexigens*.
- A CE of ~96% suggested that H_2_ was not utilized by a *D. acetexigens* monoculture.
- Target electroactive species showed niche dominance in mixed species anodic biofilms.
- The hypothesis of electrode-mediated ethanol fermentation is proposed.

## 1 Introduction

The transition from fossil-based to carbon-neutral energy systems is generally agreed on, yet its implementation remains challenging. Renewable energy sources are a central component of this transition, but their fluctuating availability requires efficient storage strategies (Fekete et al., 2023). One option is the conversion of electrical energy into chemical energy carriers, such as hydrogen. In this context, microbial electrolysis is considered a promising approach (Nevin et al., 2010). At the anode, an electroactive biofilm of microorganisms catalyzes the direct conversion of chemical energy of e.g., organic wastewater constituents into electrical energy, protons and CO_2_. At the cathode, the electrons generated reduce the protons to hydrogen. Compared to conventional methods such as abiotic water electrolysis, microbial electrolysis cells (MECs) require significantly less energy input at the cathode. This is because microbial metabolism delivers electrons at a low redox potential, thereby lowering the energy demand for hydrogen production (Zhang and Angelidaki, 2014). In order to achieve high current densities with high hydrogen yields in MECs, existing systems must be optimized (Hackbarth et al., 2023; Xiao et al., 2025). In addition to improving the reactor design or chemical functionalization of anode surfaces, the implementation of new microorganisms as biocatalysts can also increase performance (Ketep et al., 2013).

Electroactive microorganisms (EAMs) are capable of extracellular electron transfer (EET), enabling them to couple intracellular metabolic reactions to insoluble electron acceptors such as anodes. *Geobacter sulfurreducens* is such an exoelectrogenic, biofilm-forming model organism, in which the formation of type IV pili (Reguera et al., 2006), together with a high abundance of c-type cytochromes (Methé et al., 2003) have been described. In pure culture, *G. sulfurreducens* is capable of forming anodic biofilms with a thickness exceeding 160 μm (Leang et al., 2013).

Several studies have reported the co-occurrence of *Desulfuromonas acetexigens* with *G. sulfurreducens* in various anodic biofilms enriched from mixed inocula, including anaerobic sludge (Sapireddy et al., 2021), domestic wastewater (Ketep et al., 2013), raw paper mill effluents (Ketep et al., 2013), and lagoon sediments (Ishii et al., 2014). Katuri et al. (2017, 2020) demonstrated that a pure culture of *D. acetexigens* was capable of generating high peak current densities of ~10 A m^−2^ at the anode of an MFC supplied with 10 mM acetate. In a direct comparison of bioelectrical efficiency, *G. sulfurreducens* achieved a maximum current density of ~8 ± 0.5 A m^−2^, while *D. acetexigens* produced 11 ± 0.1 A m^−2^ under identical cultivation conditions (Sapireddy et al., 2021).

A clear drawback of using *G. sulfurreducens* in MECs is its utilization of hydrogen as an electron donor (Speers and Reguera, 2012; Knoll et al., 2023). This is reflected in the Coulombic efficiency (CE) for *G. sulfurreducens* biofilms in MECs. In the study of Sapireddy et al. (2021), *G. sulfurreducens* achieved a CE of 110 ± 10%, whereas *D. acetexigens* exhibited a CE of 98 ± 2%. Thus, the lack of H_2_ utilization by *D. acetexigens* results in a higher hydrogen yield (Finster et al., 1994; Katuri et al., 2020).

Although *D. acetexigens* has gained increasing attention in bioelectrochemical research, its biofilm morphology under chronoamperometric cultivation conditions has not yet been systematically investigated. In particular, the relationship between biofilm morphology and electrochemical performance of *D. acetexigens* remains unclear. One approach to assess this structure—function relationship is biofilm imaging using optical coherence tomography (OCT). OCT is a non-invasive imaging technique that enables online visualization of native biofilms (without staining). It provides access to various morphological parameters, such as biofilm thickness, structural heterogeneity, or substratum coverage. The acquisition of OCT data during reactor operation also offers statistically robust evaluation of biofilm development (Wagner and Horn, 2017). In this context, this study investigates the biofilm development and the chronoamperometric behavior of *D. acetexigens* in direct comparison to *G. sulfurreducens*. Biofilms were cultivated on planar graphite anodes in flow cell MECs. By combining OCT with the electrochemical measurements, insights were gained into how the biofilm architecture influences and potentially limits its performance. Furthermore, the impact of contaminants on substrate turnover and coulombic efficiency was analyzed, leading to the discussion of a potential metabolic interaction between the contaminant *Sporomusa sphaeroides* and the EAM.

## 2 Material and methods

### 2.1 Strains and preculture conditions

*Desulfuromonas acetexigens* 2873 (DSM 1397) and *Geobacter sulfurreducens* PCA (DSM 12127) were both activated as lyophilized cultures as described by the German Collection of Microorganisms and Cell Cultures (DSMZ, Germany). After activation, both strains were pre-cultured separately at 30 °C under anoxic conditions in 250 mL airtight, rubber septa-sealed anaerobic bottles, containing 200 ml BES-Medium with a pH = 7.4, buffered with 25 mM HEPES buffer. The detailed composition of this minimal medium can be found elsewhere (Knoll et al., 2022; Xiao et al., 2025). Briefly, 20 mM sodium acetate and 40 mM of disodium fumarate were added as electron donor and acceptor, respectively. After precultivation, both cultures were centrifuged for 10 min at 4,600 × *g* and 4 °C (Rotana 460 R, Hettich, Tuttlingen, Germany) in previously autoclaved centrifuge tubes (BN: 0512; Hettich, Tuttlingen, Germany) and mid-log phase cells were harvested and washed in sterile saline solution (0.44 g L^−1^ KH_2_PO_4_; 0.22 g L^−1^ K_2_HPO_4_; 0.2 g L^−1^ NH_4_CL; 0.38 g L^−1^ KCL; 0.36 g L^−1^ NaCl) to remove fumarate. The pellets were then resuspended in 20 mL of the sterile saline solution.

### 2.2 Flow cell setup

**Figure 1:**
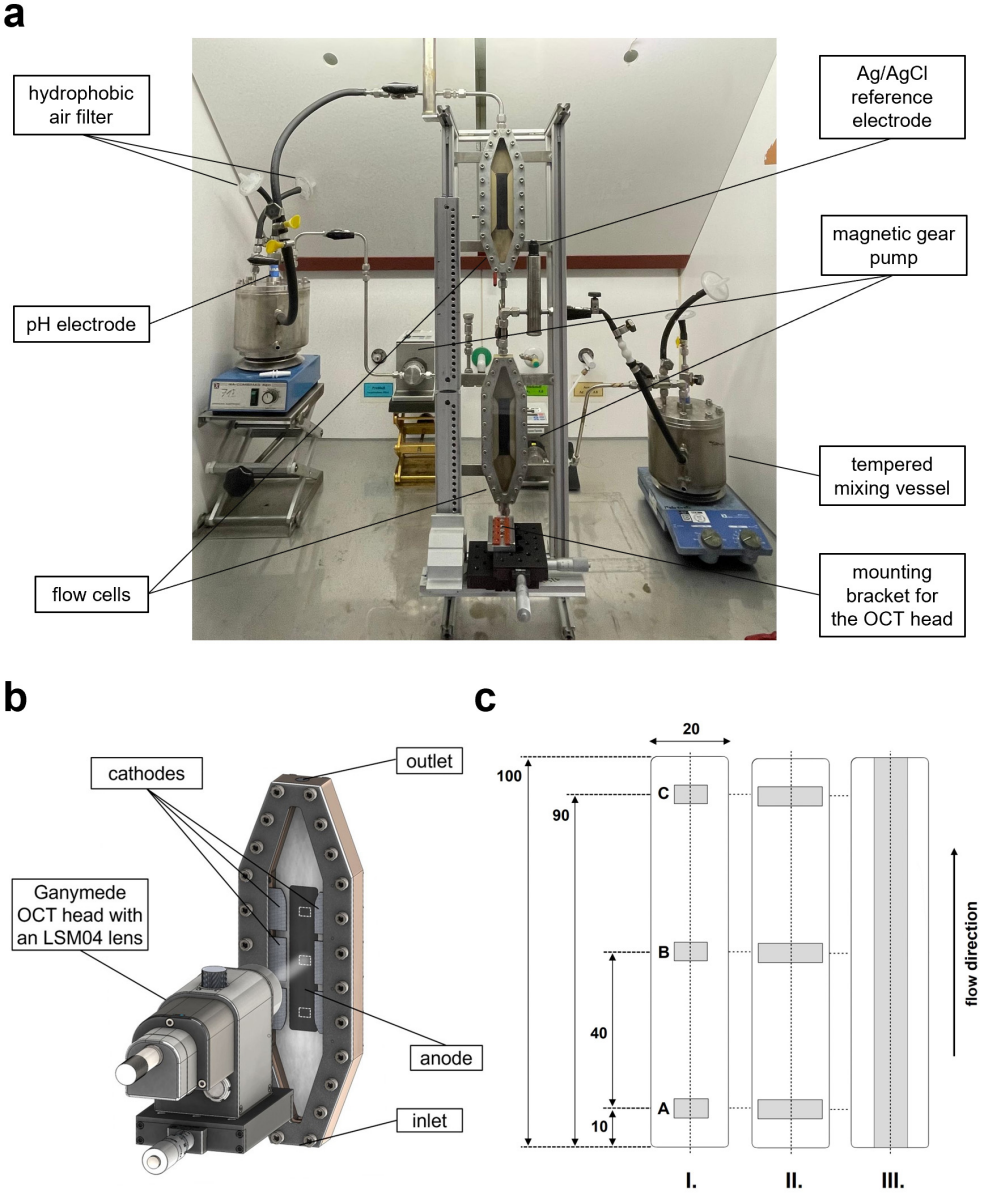
Illustration of the microbial electrolysis setup. **(a)** photograph of two separate, vertically positioned flow cells plus peripheries, with the upper cell being connected to the periphery on the left and the lower cell to the one on the right. The holder for the OCT head is located in front of the flow cell channel, which can be adjusted to precise positions at vertical intervals of 1 cm. This allows the entire length of the anode to be visualized. **(b)** 3D rendering of a flow cell; view of the anodic and cathodic electrodes. The white squares indicate the three OCT recording positions [A, B, C; 8 × 6 mm (W × L)] on the anode (20 cm^2^). **(c)** Positions of the areas (indicated in gray) visualized via OCT on the anode (white). Dimensions are given in mm. The respective center points of A, B and C lie on the horizontal symmetrical axis of the anode (dashed line). The flow direction is indicated. Measurement scenarios (I): Each area (A, B, and C) measures 8 × 6 mm^2^ (W × L). (II): Each area (A, B, and C) measures 15 × 6 mm^2^ or 16 × 6 mm^2^ (W × L), comprised of two adjacent or slightly overlapping single images. (III): The visualized area measures 8 × 100 mm^2^ (W × L).

The bioelectrochemical flow cell applied in this work was developed by Max Hackbarth to ensure constant cultivation conditions and enable the use of online *in-situ* monitoring tools, like optical coherence tomography for visualization of biofilm structure Section 2.7 (Hackbarth et al., 2020). Figure 1 shows the Illustration of the flow cell setup according to Hackbarth et al., 2020. In contrast to previous work, the flow cell was positioned vertically (denotation E in Figure 1a) with the liquid entering the cell at the bottom and leaving at the top in order to avoid passive deposition of planktonic cells on the anode surface through sedimentation. This vertical positioning also prevented particle sedimentation on the anode, ensuring the biofilm formation and development was not impaired by enhanced abrasion. Furthermore, it prevented produced hydrogen bubbles from accumulating in the flow cell. Figure 1b shows a 3D rendering of the flow cell with a detailed view of the anodic and cathodic electrodes.

### 2.3 Flow cell operation

Prior to each new start-up of the reactor, the dismanteled parts of the system were cleaned successively with ethanol and demineralized water. Subsequently, the reactor was reassembled and sterilized by autoclaving. Prior to filling the reactor, the anaerobised MEC culture medium [as described by (Sapireddy et al., 2021) (pH 7.4, 10 mM sodium acetate as electron donor)] was inoculated with 3% (v/v) of the cell suspension of either *D. acetexigens* or *G. sulfurreducens* prepared as defined in Section 2.1. This corresponds to cell concentrations in the inoculated medium of 8 × 10^7^ to 3 × 10^8^ cells mL^−1^ (OD_600_ = 0.1–0.35). Only in the *D. acetexigens* duplicate cultivation Des5 (see Table 1), cultures were directly inoculated using 1 mL of cryostock solution with a glycerol content of 35% (v/v). Also, the flow cell and periphery were anaerobised by flushing the system with 100% N_2_ for 24 h to remove residual oxygen. The medium was recirculated at a volumetric flow rate of 100 mL min^−1^. This resulted in a mean flow velocity of ν = 1.5 cm s^−1^ at a distance of 500 μm above the anode (Hackbarth et al., 2020). Temperature was controlled to 30 °C by the double-walled mixing vessel which was connected to a circulating thermostat (Dyneo DD, Julabo Seelbach, Germany). By means of a potentiostat (Interface 5000P, Gamry Instruments, Warminster, USA), a constant potential of 0 mV vs. SHE (standard hydrogen electrode) was maintained at the anode for the duration of a single experiment (of 9–36 days) while recording the current densities measured. An exception is the chronoamperometric analysis, Des1 (see Table 1), whose procedure is described in the Supplementary material. Uninoculated flow cell operations served as negative controls. Before each chronoamperometric measurement, the uncompensated resistance (Ru) between working and reference electrodes was determined. If the Ru value exceeded 8 Ohm, the electrical connections between the electrodes and the potentiostat were inspected. In all experiments, the plain graphite working electrode (20 cm^2^; MR40, Müller und Rössner, Troisdorf-Bergheim, Germany) functioned as anode and the 6 V4A-stainless steel counter electrodes (20 cm^2^) as cathode.

**Table 1:** Overview of the microbial electrolysis experiments of this study carried out in the flow cell. Inoculated bacterial strain, applied anode potential, and the inoculated cell density are provided. The respective designations of each cultivation comprise the first three letters (abbreviation) of the cultivated organism, the following number identifies the experimental series, and duplicates of experiments additionally have the suffix A or B.

**Bacterial strain**	**Designation**	**Anodic potential (mV vs. SHE)**	**Cell density (OD_600_)**
*D. acetexigens*	Des1 Des2A Des2B Des3A Des3B Des4 Des5A Des5B	−100; 0; +100 0 0 0 0 0 0 0	0.1 0.15 0.15 0.35 0.35 0.12 Cyrostock Cyrostock
*G. sulfurreducens*	Geo1A Geo1B	0 0	0.35 0.1

### 2.4 Calculations

For the evaluation and comparison of the bioelectrochemical experiments conducted in this study (see Table 1), the current density (*J*) and the Coulombic efficiency were used.

*J* (μA cm^−2^) was calculated as the ratio of the measured current (*I*) and the area of the anode *A*_*Anode*_ (20 cm^2^; Equation 1).


$$\begin{array}{l} J=\;\frac{I}{A_{Anode}} \end{array}$$ (1)

By integrating the current density over the operating period *t* of a given experiment, a mean current density ($\bar{J}$) was calculated (Equation 2). The time interval *t* starts with the onset of positive current production, marking the point after polarity reversal. At this point, the working electrode, previously involved in residual oxygen reduction, begins to perform as anode.


$$\begin{array}{l} \bar{J}\;=\;\frac{J\left(t\right)dt}{t} \end{array}$$ (2)

The evaluation of anodic oxidation was carried out by calculating the Coulombic efficiency (*CE*) according to Rabaey et al. (2005). The *CE* represents the ratio between the measured current (*I*) and the electrons released through metabolism. It thus indicates how many of the theoretically released electrons during the catalytic conversion of a substrate are recovered as current, providing insights into the bioelectrochemical efficiency of the respective process. The calculation was performed according to Equation 3:


$$CE=\frac{\int_{t_{0}}^{t}I}{c^{*}V_{R}^{*e-*}\varepsilon^{*}N_{A}}$$ (3)

with the acetate concentration consumed during that time interval (*c*, mM), the reactor liquid volume (*V*_*R*_, L), the number of electrons released during the complete oxidation of 1 mol acetate to CO_2_ (e^−^ = 8), the elementary charge (ε = 1, 602*10^−19^*C*), and Avogadro's constant (*NA* = 6, 022*10^23^ *mol*^−1^). To determine this value, the total current produced during the considered operating period *t* was obtained by integrating the measured current (*I*) (*t*_0_ = anode repolarization; *t*_end_ = final acetate sampling; the respective time window is described in detail in Supplementary material).

### 2.5 Electrochemical cultivations

In several experiments, biofilm formation and conversion performance after monoculture inoculation of the target organisms (*D. acetexigens* or *G. sulfurreducens*) with different initial cell densities were analyzed over periods of 10–38 days. An overview of all experiments conducted is provided in Table 1.

### 2.6 Analytical methods

#### 2.6.1 Ion exchange chromatography

Volatile fatty acids (formate, acetate, propionate, butyrate, isobutyrate, valerate) and lactate were quantified via ion chromatography using a Metrosep Organic Acids 250/7.8 column. Samples were sterile-filtered (0.22 μm, polyethersulfone, Lab Logistic Group, Meckenheim, Germany) and diluted 1:7.5 with deionized water to ensure concentrations within the calibration range (0.5–100 mg L^−1^). Sample volumes of 2.5–5 mL were analyzed.

#### 2.6.2 Gas chromatography

Alcohols (methanol, ethanol, 2-propanol) were measured by gas chromatography with flame ionization detection (FID). Samples were incubated in saturated NaCl solution at 90 °C, and concentrations were calculated using ChemStation software based on substance-specific retention times. Due to possible FID-associated deviations (3–10%), all measurements were performed in duplicate using a total volume of 21 mL.

#### 2.6.3 Cell quantification

Optical density (OD_600_) of bacterial cultures was measured at 600 nm using a photometer (Spectronic 1201 photometer, Milton Roy, Rodgau, Germany) with samples diluted to OD_600_ ≤ 0.5 prior to measurement.

The cell count of planktonic cultures was determined using the NovoCyte^®^ flow cytometer (Agilent, Waldbronn, Germany) and the NovoExpress software (Agilent, Waldbronn, Germany), in accordance with the manufacturer's protocol. To ensure thorough mixing and incubation of cells with the fluorescent dye SYBR Green (Invitrogen, Thermo Fisher Scientific, Karlsruhe, Germany) at 37 °C, a ThermoMixer^®^ C (Eppendorf, Hamburg, Germany) was used.

### 2.7 Biofilm characterization

**Figure 2:**
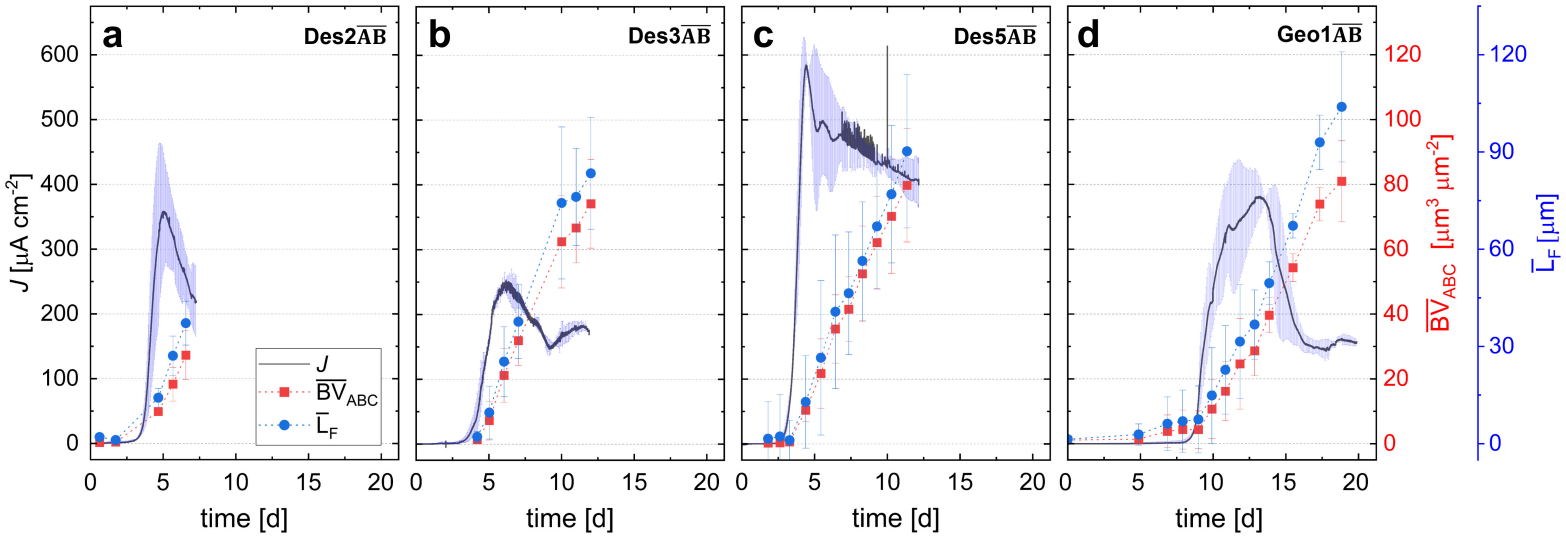
Mean measured parameters of current density and biofilm morphology in the duplicate cultivations Des2$\bar{AB}$, Des3$\bar{AB}$, and Des5$\bar{AB}$ of *D. acetexigens* and Geo1$\bar{AB}$ of *G. sulfurreducens* at an applied potential of 0 mV vs. SHE. The mean values of current density (J), average biovolume ($\left({\bar{BV}}_{ABC}\right)$, and mean biofilm thickness (${\bar{L}}_{F}$) are shown. The displayed cultivation time corresponds to the shorter experiment of the duplicate. **(a)** Mean values of duplicate Des2$\bar{AB}$, inoulated at OD_600_ = 0.15. **(b)** Mean values of duplicate Des3$\bar{AB}$, inoculated at OD_600_ = 0.35. **(c)** Mean values of duplicate Des5$\bar{AB}$, inoculated with 1 mL cryostock culture. **(d)** Mean values of duplicate Des5$\bar{AB}$, inoculated with different cell densities (Geo1A: OD_600_ = 0.15; Geo1B: OD_600_= 0.35).

The electroactive biofilm in the flow cells was visualized using the GANYMEDE II spectral domain OCT system equipped with the LSM04 lens (Thorlabs GmbH, Dachau, Germany). For all experiments, OCT images of the anode (20 × 100 mm, W × L) were recorded prior to bacterial inoculation as negative controls. Unless stated otherwise, three-dimensional OCT scans (C-scans; Wagner and Horn, 2017) were acquired at regular intervals after inoculation at three defined positions (A, B, C) along the anode, in order to evaluate biofilm formation in relation to flow velocity gradients, as described by Hackbarth et al. (2020; see Figures 1b, 2). Position A was located 1 cm above the lower flow edge of the anode; Position B was placed 4 cm above Position A at the anode midpoint; and Position C was located 1 cm below the upper flow edge of the anode (Figure 1c). The positions were adopted from Hackbarth et al. (2020), as it was demonstrated for *Kyrpidia spormannii* biofilms that the mean value of morphological characteristics (biovolume, biofilm thickness, substratum coverage) determined at these three positions represents the mean biofilm characteristics of the entire electrode area with sufficient accuracy.

OCT imaging resulted in three-dimensional datasets with dimensions of 8 mm × 6 mm × 1.75 mm (W × L × H), with a pixel resolution of 2.06 μm px^−1^ (axial, z-direction) and 8 μm px^−1^ (lateral, xy-plane), which corresponds to a visualized anode area of 48 mm^2^. To validate the representativeness of positions A–C of the anode area under the cultivation conditions of this study (Figure 1c), additional OCT scans with extended area (15–16 mm × 6 mm × 1.5 mm; W × L × H), enabling visualization of biofilm growth across up to 80% of the anode width were performed. For imaging the full length of the working electrode, 11 individual scans with a width of 8 mm each were recorded, following Hackbarth et al. (2020). Results of this validation confirm the applicability of this approach and are presented and discussed in Supplementary Figures S3–S5. The subsequent data processing and analysis was performed using ImageJ based on protocols by Wagner and Horn (2017), Bauer et al. (2019), and Hackbarth et al. (2020, 2023). During image analysis, signals caused by the biofilm had to be separated from background signals (water phase, planktonic particles, artifacts**)**. To generate comparable data, raw datasets were always binarized following the same procedure. Binarization is performed by applying a fixed threshold value of 97 consistently to all OCT datasets presented in this work to avoid manual bias and variability. This threshold value was initially derived by analyzing several C-scans using the AutoThreshold function with the “Otsu” method (Otsu, 1979; *n* = 10, threshold value = 97 ± 6). For further characterization of the biofilm structure, a total of five parameters were calculated: the biovolume *BV*, the average Biovolume $\bar{BV}$, the mean biofilm accumulation rate ${\bar{BV}}^{*}$, the mean biofilm thickness ${\bar{L}}_{F}$, and the substratum coverage *SC*.

The volume of the biofilm *V*_*BF*_ at each position was determined by counting the pixels assigned to the biofilm in the binary files and multiplying this number by the volume of the corresponding voxel. The Biovolume *BV* (μm^3^ μm^−2^) (Equation 4) is defined as the volume of the biofilm *V*_*BF*_ normalized to the monitored area *A*_*M*_:


$$\begin{array}{l} BV=\frac{V_{BF}}{A_{M}} \end{array}$$ (4)

The average biovolume $\bar{BV}$ is defined according to Equation 5:


$$\begin{array}{l} \bar{BV}=\;\frac{\sum BV_{n}}{N} \end{array}$$ (5)

Where *n* denotes the individual location/scan and *N* equals the number of the recorded data sets (*N* = 3 for positions A, B, C; *N* = 11 for the entire anode).

The mean biofilm accumulation rate ${\bar{BV}}^{*}$ (μm^3^ μm^−2^ d^−1^) is defined as the change in mean biovolume over time:


$$\begin{array}{l} {\bar{BV}}^{*}=\;\frac{\partial \bar{BV}}{\partial t} \end{array}$$ (6)

For each scan, the biofilm thickness (*L*_*F,i*_) was also determined using ImageJ. Based on this, in addition to the height profile, the height distribution, the mean biofilm thickness ${\bar{L}}_{F}$ (μm) (Equation 7) and the substratum coverage *SC* (Equation 8) were calculated according to Wagner and Horn (2017):


$$\begin{array}{l} {\bar{L}}_{F}=\;\frac{1}{N}\;\overset{N}{\underset{i=1}{\sum }}L_{F,i} \end{array}$$ (7)

Here, *L*_*F,i*_ (μm) represents the vertical distance between the anode surface and the interface between the biofilm and the bulk liquid phase at position *i* (local biofilm thickness; Okkerse et al., 2000). *N* is the number of measurements and, for a complete C-scan, corresponds to the total number of A-scans (Wagner and Horn, 2017).

The substratum coverage *SC* [%] represents the ratio of the anode surface covered by biofilm to the total monitored area:


$$\begin{array}{l} SC=\frac{A_{M}-\;A_{uc}}{A_{M}} \end{array}$$ (8)

With *A*_*uc*_ as the uncovered anode surface at the time of measurement, with the biofilm thickness ${\bar{L}}_{F}$ = 0. The lowest 2.06 μm (corresponding to the height of one pixel) above the measured anode surface were truncated in order to reduce potential distortions of the determined degree of coverage by anode irregularities.

### 2.8 Phylogenetic characterization

Illumina sequencing was used for phylogenetic characterization. The sample material (detailed description can be found in Supplementary Table S1) was sent to the private Institute for Molecular Analysis Karlsruhe GmbH (IMA, Karlsruhe, Germany) for analysis of microbial diversity (16S rDNA amplicon sequencing). This included DNA isolation, 16S rDNA PCR on the isolated gDNA, indexing, and purification of the PCR product as well as qualitative analysis. After amplicon sequencing (2 × 250 bp, min. 50,000 reads), bioinformatic evaluation and phylogenetic classification were performed using the SILVIA 16S database.

## 3 Results and discussion

### 3.1 Chronoamperometric behavior and development of *D. acetexigens* and *G. sulfurreducens* biofilms

The first part of this study focuses on the bioelectrochemical cultivations of *Desulfuromonas acetexigens* and *Geobacter sulfurreducens* and the comparison of biofilm formation, morphology, and current production. The second part addresses substrate turnover and aims to explain the utilization of unexpected electron and carbon sources in the system through taxonomic classification of the microbial community (Section 3.2) and a detailed analysis of possible interspecies metabolic interactions (Section 3.3).

As a control, a non-inoculated flow cell was operated under identical conditions. No substrate consumption, biofilm formation, or current generation was detected (see Supplementary Figure S2), confirming that the observed electrochemical activity in inoculated systems originated exclusively from electrochemical microbial catalysis. To ensure that all OCT-derived biofilm morphology data presented in the following sections are representative and statistically robust, the applied imaging routine was validated with respect to both the longitudinal and lateral heterogeneity of the anode (see Supplementary material). In addition, uniform binarization thresholds and identical data processing macros were applied across all OCT datasets, minimizing user bias and ensuring comparable morphology data.

#### 3.1.1 Anodic *D. acetexigens* cultivation

Several studies demonstrated that *D. acetexigens* does not utilize hydrogen as an electron donor (Sapireddy et al., 2021; Guo et al., 2021; Joshi et al., 2021). The 10-day chronoamperometric cultivation Des1 (see Supplementary Figure S2) confirmed this observation: from acetate consumption and current production, a coulombic efficiency of ~96%was calculated (further details are provided in Supplementary material). This indicates that~ 96% of the electrons theoretically gained from the oxidized acetate were transferred to the anode and ~4% were directed to biomass or extracellular polymeric substances (EPS). Based on the observed current densities in Des1 ($\bar{J}$ = 124.3 μA cm^−2^), the expected hydrogen accumulation rate in the gas phase was low (< 0.5 vol % h^−1^), rendering hydrogen quantification highly uncertain. Hydrogen production was therefore not quantified. Thus, this study focuses on Coulombic efficiency as a robust and integrated indicator of electron recovery.

To characterize the biofilm morphology of *D. acetexigens* and assess its impact on current generation, the relationship between average biovolume ($\bar{BV}$), mean thickness (${\bar{L}}_{F}$), and current density was investigated. For this purpose, average values of the duplicate cultivations Des2$\bar{AB}$, Des3$\bar{AB}$, and Des5$\bar{AB}$ were plotted over cultivation periods of up to 20 days (Figures 2a–c, respectively). Inoculation procedures and/or initial cell densities differed between cultivations, with optical densities (OD_600_) of 0.15 and 0.35 for Des2$\bar{AB}$, Des3$\bar{AB}$, respectively, inoculated from pre-cultures, whereas Des5$\bar{AB}$ was inoculated directly with a cryostock vial (see Table 1). Figure 2d additionally displays the chronoamperometric behavior and biofilm morphology of the model organism *G. sulfurreducens* (cultivation Geo1$\bar{AB}$), the corresponding discussion can be found in Section 3.1.2.

A striking difference among the Des-duplicates was the maximum mean current density (${\bar{J}}_{\max}$) achieved. After ~5 days, Des2$\bar{AB}$ reached a ${\bar{J}}_{\max}$ of 356 μA cm^−2^, whereas Des3$\bar{AB}$ reached 248 μA cm^−2^ after ~6 days. In Des2$\bar{AB}$ and Des5$\bar{AB}$, a decline in current density occurred once the mean biofilm volume exceeded 10.0 ± 1.2 μm^3^ μm^−2^ and 12.6 ± 4.1 μm^3^ μm^−2^, respectively, while in Des3$\bar{AB}$ this decline was only observed above ~21 ± 4 μm^3^ μm^−2^. ${\bar{J}}_{\max}$ of 585 μA cm^−2^ was achieved in the duplicate cultivation Des5$\bar{AB}$. Due to the inoculation with 1 mL cryostock culture, the Des5$\bar{AB}$. cultivations contained ~4.5 mM glycerol (0.35 g), which likely served as an alternative and well-suited electron donor for *D. acetexigens* (Shaw et al., 2025).

Comparison of mean biofilm thickness (${\bar{L}}_{F}$) and biovolume $\left(\bar{BV}\right)$ across all six cultivations showed that the average biovolume was consistently lower than the mean biofilm thickness. This difference results from the calculation methods, where biovolume includes only voxels assigned to the biofilm matrix based on Otsu thresholding (Otsu, 1979), thereby excluding voids and gaps within the structure. In contrast, mean thickness is determined from the actual biofilm height above the substratum (the uppermost detected voxel in each A-scan), independent of underlying voids. As biofilms generally exhibit a certain porosity, mean thickness was expected to exceed biovolume (Wagner and Horn, 2017).

**Figure 3:**
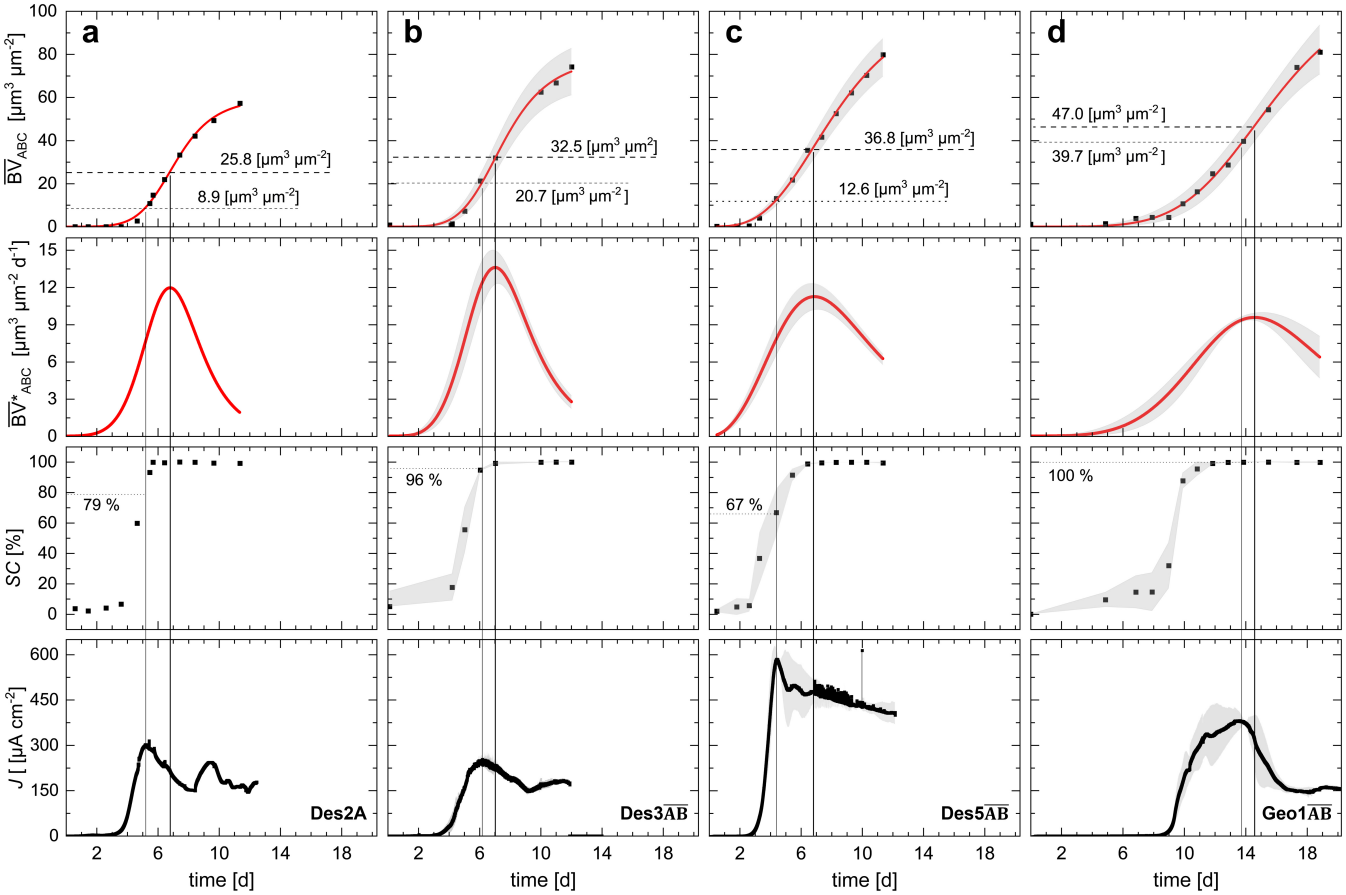
Correlation between averaged biovolume (${\bar{BV}}_{ABC}$), accumulation rates (${{\bar{BV}}^{*}}_{ABC}$), substratum coverage *SC*, and current densities (*J*) of Des2A **(a)**, Des3$\bar{AB}$ **(b)**, Des5$\bar{AB}$ **(c)** and Geo1$\bar{AB}$ **(d)**. ${\bar{BV}}_{ABC}$, ${\bar{BV}}_{ABC}$, *SC*, and current density of the replicates Des3$\bar{AB}$, Des5$\bar{AB}$, and Geo1$\bar{AB}$ (*n* = 2) are shown as mean values, derived from the respective cultivations. Shaded areas represent the corresponding ranges between measured minima and maxima.

All three *D. acetexigens* duplicates showed an onset of current production (exponential increase in current density) about 3–3.5 days after inoculation. Since the duplicates were inoculated with different cell densities, it can be concluded that the inoculated cell quantity did not affect the duration of the adhesion phase of *D. acetexigens* biofilms. After surpassing ${\bar{J}}_{\max}$, a decline in the biofilm accumulation rate (${\bar{BV}}^{*}$) would be expected, corresponding to the so-called inflection point, which marks a transition in the biofilm growth process (Hackbarth et al., 2020). To analyze this relationship between biofilm morphology and current density, Figure 3 compares mean biovolume ($\bar{BV}$), accumulation rate (${\bar{BV}}^{*}$), substratum coverage (*SC*) and current density for Des2A, Des3$\bar{AB}$, Des5$\bar{AB}$, and Geo1$\bar{AB}.$ In duplicate cultivations (Des3$\bar{AB}$, Des5$\bar{AB}$, Geo1$\bar{AB}$; *n* = 2), values of current, $\bar{BV}$, ${\bar{BV}}^{*}$ and *SC* are shown as mean ± range (minimum–maximum*)* except for cultivation Des2A where *n* = 1. Des2B was excluded from this analysis due to insufficient data for reliable fitting. The mean biofilm volume data (${\bar{BV}}_{ABC}$) were approximated using a non-linear Hill fitting according to Jin and Riedel-Kruse (2018). The corresponding equations, which yielded coefficients of determination of > 0.995 each, are provided in the Supplementary material. For further analysis, the time derivate of the fitted Equation was calculated (see Equation 6) to obtain the mean biofilm accumulation rate (${\bar{BV}}_{ABC}^{*}$) as described from Hackbarth et al., 2020. The resulting data derived from Figure 3 are summarized in Table 2.

**Table 2:** Specification of biofilm parameters determined in Des2A, Des3$\bar{AB}$, Des5$\bar{AB}$, and Geo1$\bar{AB}$ coinciding with maximum current density (${\bar{J}}_{\max}$) and the inflection point of the mean biofilm accumulation rate (${\bar{BV}}_{ABC}^{*}$). Time points after inoculation at which ${\bar{J}}_{\max}$ as well as the inflection point are given. Replicate cultivation (Des3$\bar{AB}$, Des5$\bar{AB}$, and Geo1$\bar{AB}$; *n* = 2) values are reported as mean ± range (minimum–maximum) except for cultivation Des2A where *n* = 1.

**Cultivation**	**Reference parameter**	***n***	**Time (d)**	$\bar{J}$ (μA cm^−2^)	${\bar{BV}}_{ABC}$ (μm^3^ μm^−2^)	${\bar{BV}}_{ABC}^{*}$(μm^3^ μm^−2^ d^−1^)	***SC* (%)**
Des2A Des3$\bar{AB}$ Des5$\bar{AB}$ Geo1$\bar{AB}$	${\bar{J}}_{\max}$	1 2 2 2	5.2 6.2 4.4 13.5	302 253 ± 26 585 ± 34 382 ± 29	8.9 20.7 ± 5.5 12.6 ± 2.6 39.7 ± 6.9	7.6 12.4 ± 1.9 7.8 ± 1.3 9.4 ± 0.2	79 96 ± >1 67 ± 15 100 ± <1
Des2A Des3$\bar{AB}$ Des5$\bar{AB}$ Geo1$\bar{AB}$	Inflection point of ${\bar{BV}}_{ABC}^{*}$	1 2 2 2	6.9 7.1 6.8 14.6	208 231 ± 24 476 ± 39 267 ± 105	25.8 32.6 ± 6.9 36.9 ± 5.6 47 ± 7.1	12.0 13.6 ± 1.3 11.3 ± 1.1 9.6 ± 0.3	100 100 ± <1 100 ± <1 100 ± <1

The peaks of the biofilm accumulation rate (inflection points) were observed after 6.8 to 7.1 days in all five cultivations. The maximum volumetric growth rates ranged from 11.3 ± 1.1 μm^3^ μm^−2^ d^−1^ (Des5$\bar{AB}$) to 13.6 ± 1.3 μm^3^ μm^−2^ d^−1^ (Des3$\bar{AB}$). Beyond these points, the biofilm accumulation rates declined. The corresponding mean biovolumes varied from around 26 μm^3^ μm^−2^ (Des2A) to 37 ± 5.6 μm^3^ μm^−2^ (Des5$\bar{AB}$). These data suggest that *D. acetexigens* biofilms exhibit reduced growth once an average biovolume of around 32 ± 5 μm^3^ μm^−2^ is exceeded. There are several possible causes for this inflection point, including (1) electron donor limitations by, e.g., substrate diffusion limitations from the bulk phase into or within the biofilm, or limiting substrate concentration in the medium as such, (2) electron acceptor limitations due to increased spatial distance from the anode and with-it limitations in electron transfer, (3) pH drops in the biofilm due to diffusion limitation of this product out of the biofilm and resulting unfavorable conditions for the bacteria, and (4) biomass loss through erosion or sloughing caused by increased shear forces which result from decreasing free cross-section of the flow channel and, thus, higher fluid velocities above the biofilm (Hackbarth et al., 2020; Xiao et al., 2025). In all cases, the inflection point coincided with complete anode coverage (*SC* = 100%), a point beyond which the biomass could only spread in the z-direction, perpendicular to the substratum. The biofilm morphology determined at the point of maximum current density differed slightly between all cultivations. The most effective Biovolume lays in a range of approximately ${\bar{BV}}_{ABC}$ ≈ 10–20 μm^3^ μm^−2^. Des2A and Des5$\bar{AB}$. achieved maximum current at relatively small biovolumes of 9 to 13 ± 3 μm^3^ μm^−2^ after ~5 days, whereas Des3$\bar{AB}$. required a larger biovolume (~21 ± 6 μm^3^ μm^−2^) to reach maximum current generation after ~6 days. Similarly, in Des1 (+100 mV vs. SHE) a decline in current generation occurred at ~25 μm^3^ μm^−2^ biovolume after ~6 days (see Supplementary Figure S2). Despite the inherent variability of biological systems, a distinct threshold for the “most efficient” electrocatalytic *D. acetexigens* biovolume of ${\bar{BV}}_{\bar{J}\max}$ ≈ 16 ± 6 μm^3^ μm^−2^ can be approximated.

#### 3.1.2 Anodic *G. sulfurreducens* cultivation

To examine the correlation between biofilm morphology of *G. sulfurreducens* and the generated mean current density, Figure 2d presents the averaged results of biovolume and biofilm thickness of Geo1$\bar{AB}$, plotted over cultivation time. Analysis of the mean values from both *G. sulfurreducens* chronoamperometric cultivations revealed a lag phase of ~8 days between inoculation and the onset of current generation. Since the inoculated cell densities differed (Geo1A: OD_600_ = 0.15; Geo1B: OD_600_= 0.35), the adhesion phase in the flow cells appears independent of inoculum size. In both experiments, maximum current densities (412 μA cm^−2^ in Geo1A; 386 μA cm^−2^ in Geo1B) coincided with complete substratum coverage (*SC* = 100%; Figure 3d, Table 2), unlike in the cultivations of *D. acetexigens*, where ${\bar{J}}_{\max}$ was reached before the substratum was fully covered (Des2A, Des3$\bar{AB}$, Des5$\bar{AB}$; see Figures 3a–c, Table 2).

In contrast to the chronoamperometric analyses of *D. acetexigens*, a decline in current density in *G. sulfurreducens* was only observed once the biovolume exceeded ~40 ± 7 μm^3^ μm^−2^ (Figure 3d, Table 2). This agrees with previous reports showing maximum electrochemical activity of *G. sulfurreducens* biofilms at thicknesses of ~20 μm (Sun et al., 2016) and ~50 μm (Reguera et al., 2006). In thicker biofilms, increased electron transport resistance progressively limits electron transfer (Sun et al., 2016). As a result, catabolically repressed cells contribute less to current generation and further hinder mass transport toward the substratum, restricting substrate availability for cells located near the anode surface. Another explanation for the ability of *Geobacter* biofilms to still reach maximum current densities despite of 14–31 μm^3^ μm^−2^ larger biovolumes compared to *D. acetexigens* could be attributed to differences in their extracellular electron transfer mechanisms. *G. sulfurreducens* possesses electrically conductive pilus-like protein complexes, so-called nanowires or e-pili, composed of highly conductive type IVa PilA monomers together with OmcC, OmcE, OmcS, and OmcZ cytochromes (Guo et al., 2021). PilA serves as the backbone of the nanowires, so that a drastic loss of conductivity was detected in *Geobacter*-ΔpilA deletion strains (Holmes et al., 2016). Although the presence of OmcE, OmcS (Katuri et al., 2017) and OmcZ (Guo et al., 2021) cytochromes, as well as sequence homology to type IV pilus-associated proteins (PilM, PilN, PilO; Xie et al., 2021), had been demonstrated in *D. acetexigens*, pilA itself appears to be absent from the genome (Katuri et al., 2017; Guo et al., 2021). These c-type cytochromes detected in *D. acetexigens* play a key role in the extracellular electron transfer of *G. sulfurreducens* (Katuri et al., 2017; Lovley and Walker, 2019). In 2009, Nevin et al. reported that conductive protein complexes (fimbriae) can also be composed solely of the c-type cytochromes OmcS and OmcZ (Nevin et al., 2009; Filman et al., 2019; Wang et al., 2019). However, their length differs markedly: nanowires in *G. sulfurreducens* can extend up to 20 μm (Reardon and Mueller, 2013), while OmcS–OmcZ fimbriae reach only a few micrometers (Guo et al., 2021). This difference may help to explain why *G. sulfurreducens* biofilms can reach volumes of ~40 μm^3^ μm^−2^ at ${\bar{J}}_{\max}$, whereas *D. acetexigens* biofilms already show indications of electron transfer limitations at ca.16 μm^3^ μm^−2^.

### 3.2 Substrate turnover and microbial diversity

**Figure 4:**
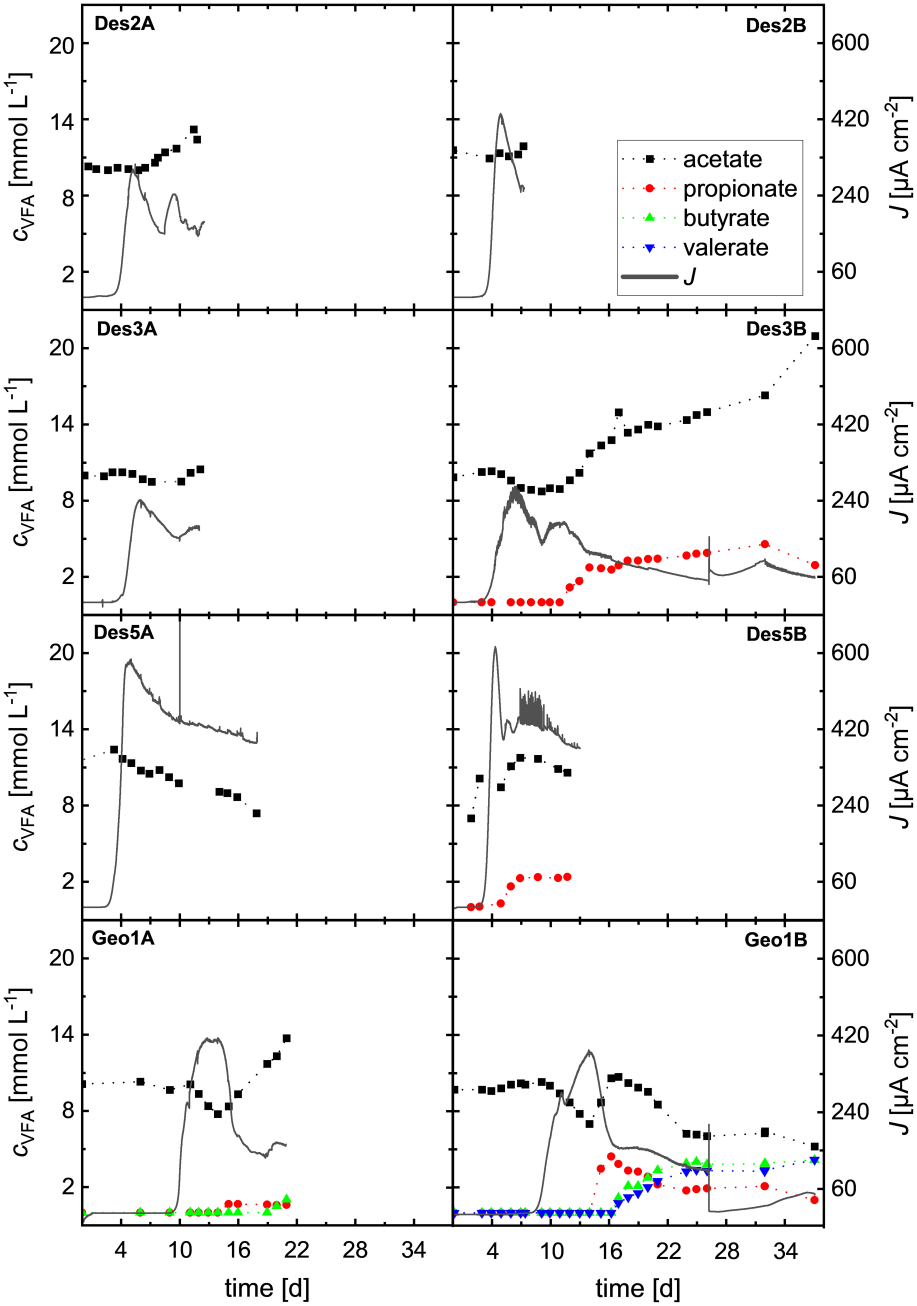
Representation of the VFA concentrations (*c*_*VFA*_) and the current density (*J*) of all duplicate cultivations.

According to Sapireddy et al. (2021), *D. acetexigens* enables higher hydrogen yields as biocatalyst of microbial electrolysis cells compared to *Geobacter sulfurreducens*. A direct comparison can be made by calculating the Coulombic efficiency (CE). However, a valid CE could only be determined in cultivation Des1 where the acetate concentration decreased by 2.2 mmol L^−1^ over the course of the experiment (Supplementary Figure S2). In all other *Desulfuromonas* as well as *Geobacter* cultivations, however, an increase in acetate concentration was observed. Figure 4 shows the development of measured concentrations of volatile fatty acids (VFA) for all duplicate cultivations of *D. acetexigens* (Des2AB, Des3AB, Des5AB), and *G. sulfurreducens* (Geo1AB), along with the corresponding current densities achieved.

From the mean current densities shown in Figure 4, the number of transferred electrons was calculated and related to the eight electrons released during complete acetate oxidation to CO_2_. This allowed estimation of a hypothetical acetate turnover, assuming acetate as the sole substrate and 100% Coulombic efficiency. The actual total electron transfer was derived from current density, with the calculation period defined from the onset of positive current (switching the working electrode to anode) until the end of cultivation. By subtracting the net acetate consumption calculated from current (*C*_*Ac, cons*_) from the initial acetate concentration *C*_*S, t*0_ the expected final concentration at the end of the experiment (*C*_*S*, exp., *tE*_) can be obtained (Equation 9).


$$\begin{array}{l} C_{Ac,\;\exp,\;tE}=C_{Ac,\;t0}-\;C_{Ac,\;cons.\;} \end{array}$$ (9)

The difference between *C*_*S*, exp., *tE*_ and the final measured concentration of volatile fatty acids (VFA) (*C*_*VFA, tE*_) can thus be attributed to a net production of VFA in the system (*C*_*VFA, prod*_; Equation 10).


$$\begin{array}{l} C_{VFA,\;prod}=C_{VFA,\;tE}-\;C_{Ac,\;\exp\;} \end{array}$$ (10)

Table 3 lists the target organisms, the respective bioelectrochemical cultivations, the time period considered for the calculations, and the mean current densities. It further summarizes the initial acetate concentration measured via ion-exchange chromatography at the start of cultivation (*C*_*Ac, t*0_), the net acetate consumption (*C*_*Ac, cons*._), the expected final acetate concentration (*C*_*Ac*, exp. *tE*_), the actually measured final VFA concentration (*C*_*VFA, tE*_), and the concentrations of fatty acids accumulated in the system (*C*_*VFA, prod*_).

**Table 3:** Representation of substrate and VFA concentrations determined by ion-exchange chromatography (C_Ac, t0_, C_VFA, tE_), the theoretically consumed acetate concentrations (C_Ac, cons. _), the expected final acetate concentration at the end of cultivations (C_Ac, exp. tE_), and the produced volatile fatty acids (C_VFA, prod_) (ac, acetate; prop, propionate; but, butyrate; val, valerate) in the cultivations Des1, Des2AB, Des3AB, Des5AB, and Geo1AB.

**Target organism**	**Cultivation**	**Time (*d*)**	**$\bar{\text{J}}$ (μA cm^−2^)**	**C_Ac,t0_ mmol L^−1^**	**C_Ac,cons._ mmol L^−1^**	***C*_Ac,exp.tE_ mmol L^−1^**	**C_VFA,tE_ mmol L^−1^**	**C_VFA,prod_ mmol L^−1^**
*D. acetexigens*	Des1	6.2	124.3	9.5	2.1 ac.	7.4 ac.	7.4 ac.	0
	Des2A	6	137.7	10.3 ac.	−1.9 ac.	8.4 ac.	12.4 ac.	4 .ac.
	Des2B	6.7	170.1	11.6 ac.	−2.5 ac.	9.1 ac.	11.9 ac.	2.8 ac.
	Des3A	12.1	115.7	10 ac.	−3.1 ac.	6.9 ac.	10.5 ac.	3.7 ac.
	Des3B	36.5	98.1	9.8 ac.	−8 ac.	1.8 ac.	21 ac. 2.9 prop.	19.2 ac. 2.9 prop.
	Des5A	17.9	359.3	10.8 ac.	−16.8 ac.	0	7.4 ac.	9.4 ac.
	Des5B	13	334.5	10.1 ac.	−12.6 ac.	0	10.6 ac. 2.4 prop.	13.1 ac. 2.4 prop.
*G. sulfurreducens*	Geo1A	12	226.4	10.1 ac.	−6.1 ac.	4 ac.	13.7 ac. 0.6 prop. 1 but.	9.7 ac. 0.6 prop. 1 but.
	Geo1B	33.9	105.1	10.3 ac.	−8 ac.	2.3 ac.	5.2 ac. 1 prop. 4.2 but. 4.2 val.	2.9 ac. 1 prop. 4.2 but. 4.2 val.

To obtain the observed mean current densities $\bar{J}$ (μA cm^−2^), up to 2.5 mmol L^−1^ acetate in duplicate Des2AB and up to 8 mmol L^−1^ in Des3AB and Geo1AB would have had to be oxidized to CO_2_ (Table 3, column 7). However, depending on the cultivation, acetate production between 2.8 and 19.2 mmol L^−1^ was detected.

The carbon balance between the start and end of cultivation also differed between the experiments. In Des3B, Des5B, Geo1A, and Geo1B, several VFAs which were not provided in the initial cultivation medium – such as propionate, butyrate, and valerate – were detected at some point. To date, propionate synthesis by *D. acetexigens* in bioelectrochemical systems (BES) has not been reported, and although formate assimilation under overflow metabolism has been described for *G. sulfurreducens* (Aklujkar et al., 2009), production of propionate, butyrate, or valerate has not been observed. These findings indicate multiple causes for the continuous VFA production observed during cultivation.

**Figure 5:**
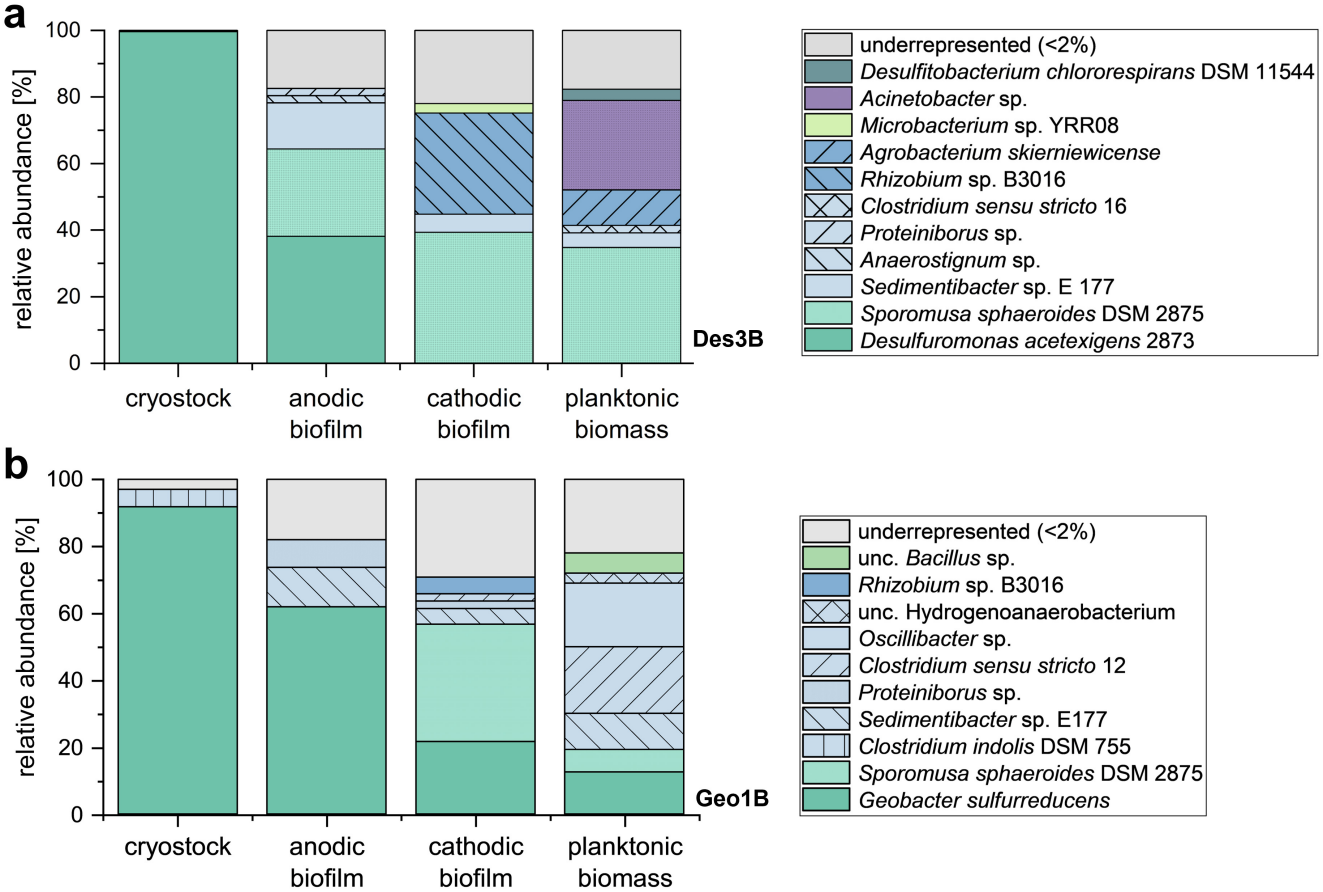
Taxonomic classification of the 16S amplicon sequencing of *D. acetexigens* cultivations Des3B and *Geobacter sulfurreducens* cultivation Geo1B at species level. Samples were taken from the cryostocks used for inoculation, the anodic and cathodic biofilms, and the planktonic cell fraction of the microbial flow cells. A representation of the taxonomically classified bacterial classes is provided in the Supplementary material (Supplementary Figure S6). **(a)** *Desulfuromonas acetexigens* cultivation Des3B. **(b)** *Geobacter sulfurreducens* cultivation Geo1A.

To assess potential microbial diversity, 16S amplicon sequencing was performed on the cryostock cultures used for inoculation, as well as on anodic and cathodic biofilms, and the planktonic microorganisms of cultivations Des3B (Figure 5a) and Geo1B (Figure 5b; Supplementary Table S1). Taxonomic classification by species is shown in Figure 5, whereas the representation of the bacterial classes is provided in Supplementary Figure S6.

The taxonomic classifications revealed a high abundance of contaminants in anodic, cathodic, and planktonic fractions. While the frozen inoculum of *D. acetexigens* represented a pure culture (> 99.5% relative abundance), the *G. sulfurreducens* stock already contained low-level *Clostridium* contamination (< 5%). This explains the high abundance of *Clostridium* in the anodic biofilm (< 20%) and planktonic biomass (< 50%) of Geo1B although its presence was also detected in Des3B. Some *Clostridium* species are known to produce diverse fatty acids in mixed-culture MECs (Atasoy and Cetecioglu, 2021; Lenin Babu et al., 2013). Also, some strains of the genus *Clostridium*, belonging to the closely related *Clostridium sensu stricto* group, have also been identified as highly abundant members of anodic biofilms in MECs where *Geobacter* spp. dominated anodic respiration. It has been suggested that certain *Clostridium sensu stricto* strains may scavenge part of the electrons transferred by anodically respiring bacteria such as *Geobacter* spp. and use them as electron donors (Rabaey et al., 2004; Rago et al., 2017). Since *Clostridium* spp. can use acetate to synthesize VFAs, they may account for the detected propionate, butyrate, and valerate (Figure 4; Lenin Babu et al., 2013; Atasoy and Cetecioglu, 2021).

In Des3B and Geo1B, unclassified *Clostridium sensu stricto* clusters were detected (Cluster-12 in Des3B; Cluster-16 in Geo1B) with relative abundances of ~2.2% in the cathodic biofilm of Geo1B and 2–20% in planktonic fractions. Since they were absent from the anodic biofilms, a direct interception of electrons from anodic EAM can be excluded (Kracke et al., 2015). In both systems, additional taxa were detected. Alphaproteobacteria of the Rhizobiaceae family (*Rhizobium* sp., *Agrobacterium skierniewicense*) dominated parts of the cathodic biofilms, though their role in MECs remains unclear. Analysis of the anodic biofilms revealed a strong dominance of the target organisms in both systems (Des3B > 38%; Geo1B > 61.5%). In Des3B, the second most abundant species was classified within the Negativicutes (26.5%), represented by *Sporomusa sphaeroides* DSM 2875. *S. sphaeroides*, a homoacetogenic and electrosynthetic bacterium with a broad substrate spectrum, was also highly enriched in cathodic biofilms (up to 39%) and present in planktonic fractions of Des3B and Geo1A.

The presence of *S. sphaeroides* likely contributed to acetate production: *S. sphaeroides* was described in several studies as a well-known organism to produce acetate from CO_2_ and H_2_ in bioelectrochemical systems (Aryal et al., 2017; Nevin et al., 2010, 2011). Members of the *Sporomusa* genus preferentially use hydrogen as electron donor with CO_2_ as terminal acceptor, but they can also ferment short-chain alcohols such as ethanol, propanol, or butanol to acetate (Möller et al., 1984). In MECs, anodically produced CO_2_ and cathodically generated H_2_ could therefore drive homoacetogenic acetate synthesis. (Möller et al., 1984). Its absence from the anodic biofilm of *G. sulfurreducens* indicates niche competition, where *Geobacter* directly consumes cathodically produced hydrogen due to the proximity of the anode and cathodes (Figure 1b). *G. sulfurreducens* itself was also detected in the cathodic biofilm of Geo1B, consistent with its ability to consume H_2_ as an alternative electron donor. Since *D. acetexigens* was not detected in the cathodic biofilm (relative abundance < 2%), this further supports its inability to utilize hydrogen as an electron donor (Engel et al., 2019; Edel et al., 2022; Sapireddy et al., 2021; Finster et al., 1994).

Considering all bacterial species detected in both systems, literature comparison confirmed that *D. acetexigens* and *G. sulfurreducens* were the only electroactive bacteria present in the sequenced samples. Therefore, the observed current densities can be attributed solely to their catalytic activity and extracellular electron transfer. In summary, acetate production in both systems (Des3B and Geo1B) can likely be attributed to homoacetogenesis by *S. sphaeroides* as well as VFA synthesis by *Clostridium* contaminants. Assuming acetate was the only carbon source available, however, the higher final acetate concentrations compared to the starting levels (Table 3) cannot be explained. This indicates the presence of an additional carbon and electron source in the system that was used for acetate production.

### 3.3 Alternative carbon sources

At the start of each cultivation, CO_2_ was removed by N_2_ sparging (24 h), so that the systems contained little dissolved CO_2_ The supplied carbon sources (10 mmol L^−1^ acetate, vitamins, Cas-amino acids) were insufficient to explain the observed increase in organic carbon, mainly as acetate. The most plausible additional source was ethanol (EtOH), introduced during sterile assembly via non-autoclavable components disinfected with 70% EtOH spray. Measurements indicated that a few sprays could contribute up to ~40 mmol L^−1^ (2.4 g of 70% EtOH) EtOH to the reactor. Although EtOH was not initially considered, later gas chromatography analyses detected concentrations between 1.6 and 28 mmol L^−1^ in all *D. acetexigens* and *G. sulfurreducens* cultures (Supplementary Table S1). Due to the volatility of EtOH, these values should be seen as indicative rather than absolute. Importantly, ethanol fermentation to acetate or longer-chained fatty acids had not been reported for *D. acetexigens* or *G. sulfurreducens*, suggesting that additional community members were responsible for the observed VFA formation (Viulu et al., 2013; Guo et al., 2021).

#### 3.3.1 EtOH based chain elongation

Microbial chain elongation is a known pathway of anaerobic ethanol degradation, in which ethanol is used to elongate the acyl chain of short-chain carboxylates by two carbon units per reaction step, resulting in the formation of longer-chain fatty acids (Barker and Taha, 1942; Candry et al., 2020). The chain elongation is mainly exemplified by *Clostridium kluyveri*. The process follows the stoichiometric equation shown in Equation 11; for clarity, caproate (C6) and caprylate (C8) production are not included (Buckel and Thauer, 2018).


$$\begin{array}{l} 6\;EtOH+4\;acetate^{-}\to 5\;butyrate^{-}+H^{+}+2\;H_{2}+4\;H_{2}O\\ \;\Delta G^{{}^{\circ}\prime }=-\;180\;kJ/reaction \end{array}$$ (11)

*C. kluyveri* belongs to the *Clostridium* sensu stricto group and combines ethanol fermentation with reverse β-oxidation, enabling the conversion of acetyl-CoA (C2) into longer-chain fatty acids such as butyrate (C4), caproate (C6), or caprylate (C8; Cruz-Morales et al., 2019; Fuchs et al., 2022; Spormann, 2023). These fatty acids can serve as carbon and energy sources, biomass precursors, or storage compounds (Courtney et al., 2023). Importantly, reverse β-oxidation may also act as an electron sink during overflow metabolism, with excess electrons from ethanol oxidation being secreted in the form of fatty acids (Scarborough et al., 2020; Quintela et al., 2024). As members of the *Clostridium* sensu stricto group were detected in Des3B and Geo1B, it is plausible that such bacteria contributed to the butyrate production observed in Geo1A and Geo1B (Cruz-Morales et al., 2019; Izadi et al., 2020; Usman et al., 2022).

#### 3.3.2 EtOH-to-acetate fermentation

Since all cultivations (Figure 4) revealed an tremendous acetate generation of up to 19 mmol L^−1^ (Table 3) and EtOH was most likely the only alternative carbon source, this section discusses a possible direct EtOH to acetate fermentation: The following stoichiometric Equation 12 can be assumed (Madigan et al., 2020):


$$\begin{array}{l} 2\;CH_{3}CH_{2}OH+2\;H_{2}O\to 4\;H_{2}+2\;CH_{3}COO^{-}+2\;H^{+}\\ \;\Delta G^{{}^{\circ}\prime }=\;+19.4\;kJ/reaction \end{array}$$ (12)

Under standard conditions (ΔG^°′^; 1 M, 1 atm, pH 7), the Gibbs free energy of the EtOH-to-acetate reaction is positive, making it endergonic and unable to support growth with ethanol as the sole substrate (Madigan et al., 2020) The four moles of hydrogen produced thus act as a reaction-limiting product. Therefore, natural EtOH fermentations often occur in syntrophy, e.g., of an H_2_ producing, ethanol-oxidizing bacterium and a hydrogenotrophic methanogen (Madigan et al., 2020; Du et al., 2025; Fuchs et al., 2022). H_2_ produced by the syntroph is immediately consumed together with CO_2_ for methanogenesis, keeping hydrogen at very low concentrations (Thiele et al., 1988). This maintains the reaction rate and the overall free energy of the process in the exergonic range, a phenomenon described as syntrophic hydrogen transfer (Madigan et al., 2020).

The cultivations showed a mixed-culture composition, with *S. sphaeroides* dominating the cathodic biofilm (Figure 5) which as previously described is capable of fermenting ethanol to acetate (Möller et al., 1984). In addition, acetate oxidation likely resulted in elevated CO_2_ concentrations in the medium, which *S. sphaeroides* can reduce via the reductive acetyl-CoA (Wood–Ljungdahl) pathway, using hydrogen as the preferred electron donor (Thauer et al., 1977; Möller et al., 1984; Aryal et al., 2017; Breznak, 2006) The corresponding stoichiometric equation is given in Equation 13.


$$\begin{array}{l} 2HCO_{3}^{-}\;+H^{+}\;+\;4H_{2}\;\to \;CH_{3}COO^{-}\;+\;4H_{2}O\\ \;\Delta G^{{}^{\circ}\prime }=\;-104.6kJ/reaction \end{array}$$ (13)

When ethanol fermentation and acetogenesis are combined (Equation 14), the overall reaction can be classified as exergonic:


$$\begin{array}{l} 2\;CH_{3}CH_{2}OH+2HCO_{3}^{-}\;\to \;3\;CH_{3}COO^{-}+3\;H^{+}+2\;H_{2}O\\ \;\Delta G^{{}^{\circ}\prime }=-85.6\;kJ/reaction \end{array}$$ (14)

**Figure 6:**
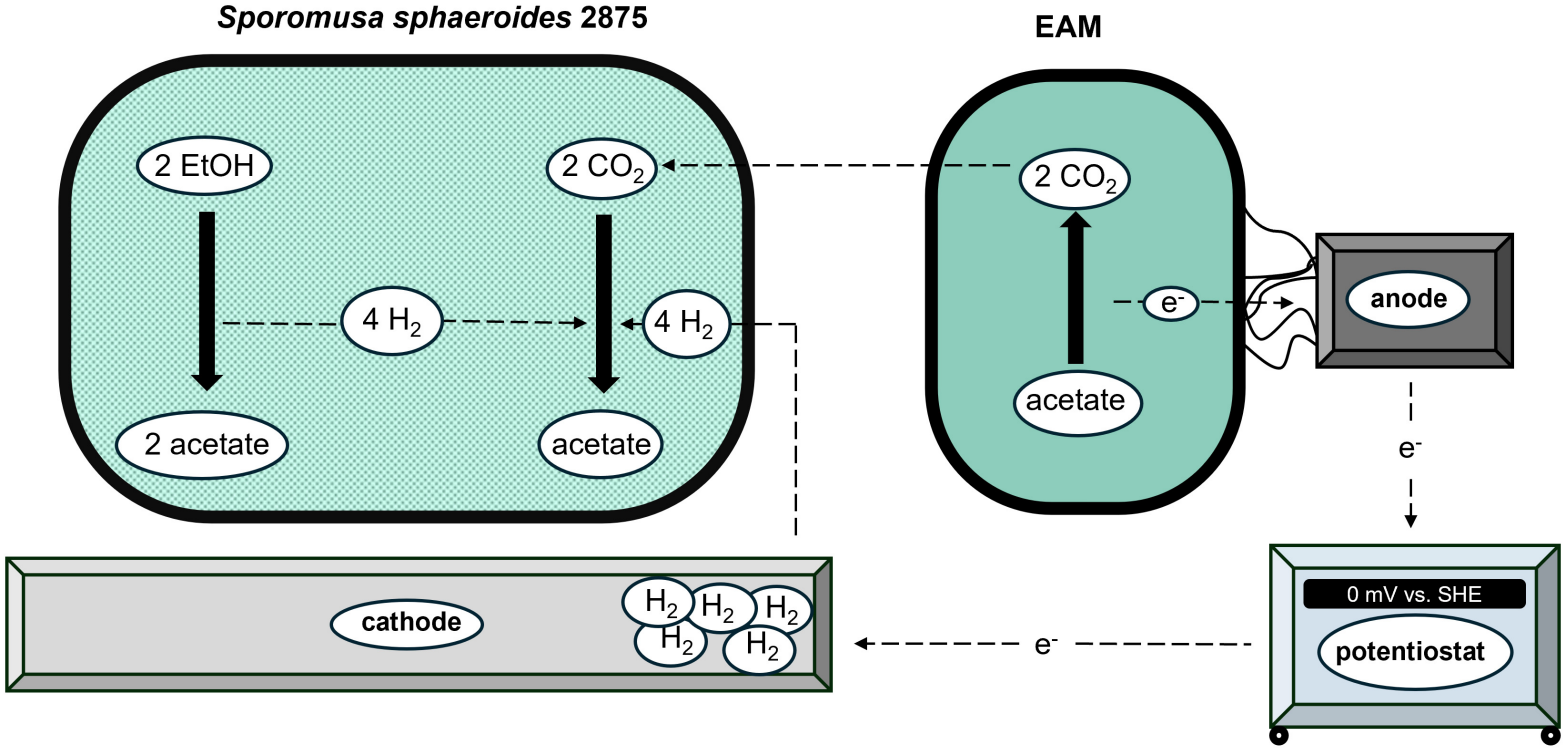
Schematic representation of the hypothesized metabolic interaction involving ethanol conversion by *Sporomusa sphaeroides* DSM 2875 and electroactive microorganisms (EAM) oxidizing acetate to CO_2_ in a microbial electrolysis cell.

To render the direct EtOH-to-acetate fermentation thermodynamically feasible, the reaction-limiting product hydrogen must be directly utilized as an electron donor for CO_2_ fixation (homoacetogenesis, Equation 14; Thauer et al., 1977; Seitz et al., 1990). As the system can be considered closed with respect to external CO_2_ supply, the CO_2_ concentration depends on the respiratory efficiency of *D. acetexigens* and *G. sulfurreducens* and only increases during cultivation. Consequently, ethanol fermentation by *S. sphaeroides* is directly linked to this CO_2_ concentration, which is influenced by the target organisms (Madjarov et al., 2022). While *S. sphaeroides* is well established in electrochemical systems, the unexpected aspect is its possible role in an electrode mediated coupling of metabolic products: the single chamber bioelectrochemical environment links hydrogen turnover (acetogenesis) and CO_2_ availability (acetate oxidation). The results suggest the hypothesis that bioelectrochemically induced interspecies metabolic interactions can occur on the basis of ethanol conversion (Summers et al., 2010). A schematic representation of the proposed overall reaction is shown in Figure 6.

This hypothesized interaction may also explain why acetate concentrations in all bioelectrochemical cultivations only began to increase after a prolonged lag phase (Figure 4). Since the cyrostock cultures did not contain *S. sphaeroides* (Figure 5), its initial abundance at reactor start-up was likely low. The delayed onset of acetate production may therefore be attributed to the adaptation and enrichment phase of ethanol-converting acetogenic community members like *Sporomusa*.

To further assess ethanol fermentation, a positive control experiment (Supplementary Figure S7; see Supplementary material for details) was conducted in which 1 ml 70% EtOH was actively supplied to cultivation Des3B (day 32) and resulted in a near-stoichiometric ethanol-to-acetate conversion of approximately 5.5 mmol L^−1^ in ~5 days. In contrast, the negative control cultivation, Des4 (Supplementary Figure S8; see Supplementary material for details), in which ethanol addition was completely omitted, showed no increase in acetate concentration.

While classical fermentative pathways, like cathodic homoacetogenesis or clostridial fermentation routes, further hydrogen recovery losses or ethanol contamination artifacts cannot be excluded, the findings support the assumption that ethanol availability, mixed-culture composition, and sustained electrochemical activity suggest the coupling of ethanol conversion and acetogenic processes in single chamber MECs.

## 4 Conclusions

This work demonstrates that *Desulfuromonas acetexigens* forms stable and well-performing biofilms on planar graphite surfaces, highlighting its strong potential as a biocatalyst for microbial electrolysis cells (MECs). Comparative cultivations with *Geobacter sulfurreducens* revealed distinct biofilm morphologies and performance patterns: while both species reached similar maximum current densities of ~420 μA cm^−2^ utilizing acetate, *G. sulfurreducens* maintained efficient electron transfer even at higher biofilm volumes (${\bar{BV}}_{{\bar{J}}_{\max}}$ ≈ 40 μm^3^ μm^−2^), whereas biofilms of *D. acetexigens* showed electron transfer limitations biofilm volumes above ${\bar{BV}}_{{\bar{J}}_{\max}}$ ≈ 16 μm^3^ μm^−2^. Notably, *D. acetexigens* displayed a markedly faster current onset (~4 vs. 8 days). Phylogenetic analyses indicated mixed bacterial communities; however, the target species were the only electroactive microorganisms detected, even after extended cultivation, suggesting niche dominance within anodic biofilms. The production of various VFAs and the spatial distribution of the involved species indicate an electrode mediated ethanol fermentation between acetogenic members of the microbial community such as the contaminant *Sporomusa sphaeroides* and the electroactive strains. In monoculture, *D. acetexigens* reached a Coulombic efficiency of ~96%, confirming that hydrogen was not consumed as an electron donor, while net increase in acetate was observed in other experimental runs. This highlights *D. acetexigens* as a promising biocatalyst for MEC applications. Compared to *G. sulfurreducens, D. acetexigens* exhibits equal or even superior electrochemical performance, reinforcing its potential for sustainable bioelectrochemical systems aimed at energy recovery and hydrogen production.

## Data Availability

The original contributions presented in the study are included in the article/Supplementary material, further inquiries can be directed to the corresponding author.
