# Supplementary material for: Relationship of electrochemical performance and biofilm development of Desulfuromonas acetexigens and Geobacter sulfurreducens in microbial electrolysis cells
Source: Front Microbiol. 2026 Mar 10;17:1753230. doi: 10.3389/fmicb.2026.1753230 (PMC13011354; doi:10.3389/fmicb.2026.1753230)
Supplement: Supplementary file 1 [file Data_Sheet_1.pdf]

Supplementary Materials:

<u>equations:</u>

Approximation of Des2A by a non-linear Hill fit (equation S1)

$$y = 59.88655 * x^{\frac{6.80737}{8.70985^{6.80737}+x^{6.80737}}} \qquad \text{(eq. S1)}$$

with a coefficient of determination (R^2) of 0.997

Approximation of Des3$\overline{\mathrm{AB}}$ by a non-linear Hill fit (equation S2)

$$y = 79.31851 * x^{\frac{5.01815}{7.61653^{5.01815}+x^{5.01815}}} \qquad \text{(eq. S2)}$$

with a coefficient of determination (R^2) of 0.995.

Approximation of Des5$\overline{\mathrm{AB}}$ by a non-linear Hill fit (equation S3)

$$y = 110.45202 * x^{\frac{3.12349}{8.51285^{3.12349}+x^{3.12349}}} \qquad \text{(eq. S3)}$$

with a coefficient of determination (R^2) of 0.997.

Approximation of Geo1$\overline{\mathrm{AB}}$ by a non-linear Hill fit (equation S4)

$$y = 115.22083 * x^{\frac{5,39741}{16.81917^{5.39741}+x^{5.39741}}} \qquad \text{(eq. S4)}$$

with a coefficient of determination (R^2) of 0.996.

Phylogenetic characterization

Table S1: Description of the sequenced samples.

Sample description	organism	experimental duplicate
cryostock culture<br>anodic biofilm<br>cathodic biofilm<br>planktonic bulk phase	*D. acetexigens*	Des3B
cryostock culture<br>anodic biofilm<br>cathodic biofilm<br>planktonic bulk phase	*G. sulfurreducens*	Geo1B

Samples of planktonic biomass as well as of anodic and cathodic biofilms, were taken after the cultivations Des3B and Geo1B were terminated (~38 days after reactor start-up). Under sterile conditions, 45 mL of bulk phase medium was withdrawn from the flow cell periphery (Figure 1a) and centrifuged for 10 minutes at 4,600 × g and 4 °C. After removal of the supernatant, further 45 mL were processed in the same way. The resulting cell pellets were stored at −80 °C until further analysis. For biofilm sampling, the liquid phase was drained from the vertically positioned flow cell. After the cover lid was removed under sterile conditions, anodic and cathodic biofilms were accessed separately. Following spatial separation of the electrodes to avoid direct contact, biofilms were scraped from the electrode surfaces using sterile 1 mL pipette tips, transferred into 2 mL microcentrifuge tubes, and stored at −80 °C. The total volume of biofilm recovered was approximately 500 µL per sample. Cryostock cultures were

centrifuged for 60 s at 16,000 × *g* (Biofuge pico, Kendro, Osterode, Germany). After removal of the supernatant, the pellets were stored at −80 °C.

EtOH concentrations

Table S2: Ethanol concentrations at different time points during the duplicate cultivations Des2AB, Des3AB, and Geo1AB after repolarization of the working electrode as the anode.

cultivation	time (d)	ethanol (mM)
Des2A	10	6.5
Des2B	6	12.5
Des3A	8	2.1
Des3B	26	1.6
Geo1A	9	2.5
Geo1B	14	28
	26	0

Chronoamperometric negative control

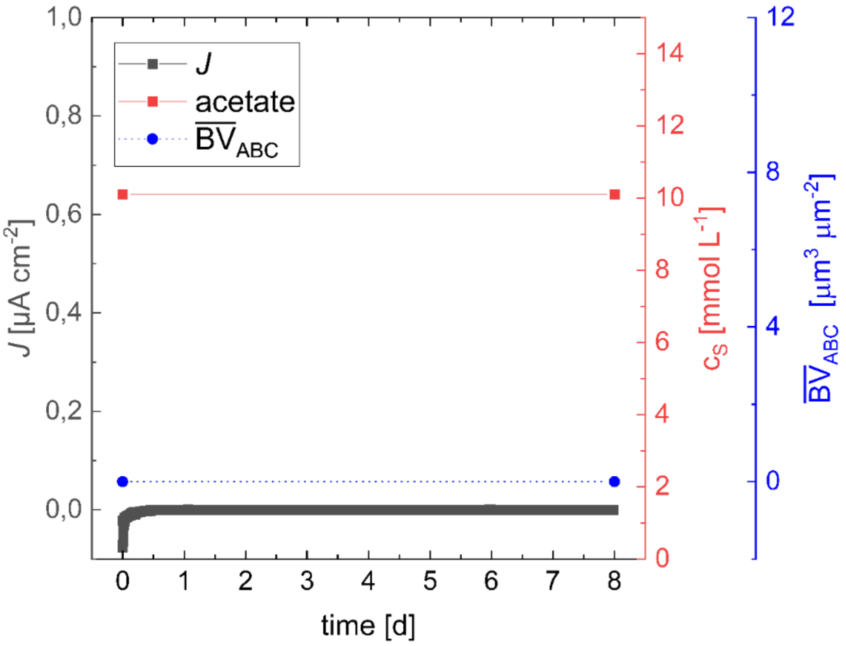

Figure S1: Representation of the negative control for all bioelectrochemical cultivations presented in this study.

Chronoamperometric cultivation: Des1

In the chronoamperometric experiment (Des1), the electrochemical properties and growth behavior of a *D. acetexigens* biofilm were analyzed at different anode potentials (Figure S2). The MEC was inoculated at a rather low initial optical density of $OD_{600} \approx 0.1$ (8×10^7 cells mL^{-1}), which had been proven to still allow for OCT imaging of the electrode surface without interference by the bulk phase optical properties (Hackbarth et al., 2023; Knoll et al., 2023). The applied MEC medium (Sappiredy et al., 2021) was supplemented with 10 mM acetate as the sole energy source (substrate concentration denoted as c_S). Biofilm morphology was visualized by OCT at 1 – 3-day intervals and quantified by image analysis.

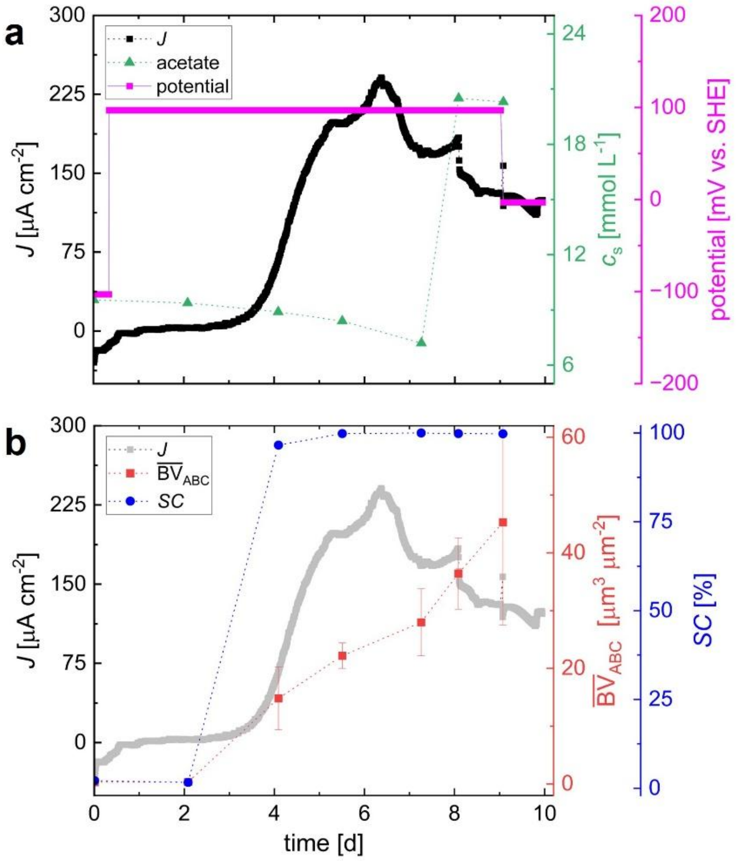

Figure S2: Measured parameters of current density and biofilm morphology in the Des1 cultivation of *D. acetexigens* at different applied anode potentials. **(a)**: The values of current density (***J***), the anodic

potential and the substrate concentration (***c_s***) are shown. **(b)**: The values of biofilm development, i.e. the average degree of anode coverage with biofilm (SC, substratum coverage) and the mean biovolume ($\overline{BV}_{ABC}$) are shown. These parameters were determined at the three recording positions (A, B, and C).

At the start of cultivation, the anode potential was set to –100 mV vs. SHE to account for negative initial currents resulting in a polarity reversal of the electrodes., caused by residual oxygen reduction (equation S5).

$$O_2 + 4e^- + 4H^+ \rightarrow 2OH^- + 2H^+ \rightarrow 2H_2O \qquad \text{(eq. S5)}$$

Within 24 h, the current density increased from –75 to 0 µA cm^{-2}, after which the potential was raised to +100 mV vs. SHE, as this value has been reported to yield high current densities for *D. acetexigens* biofilms (Katuri et al*.,* 2017 and 2020; Sapireddy et al., 2021). After ~3.4 days, the current density began to increase, reaching a maximum of 241 µA cm^{-2} after 6.5 days. This was accompanied by an acetate consumption from 9.5 to 7.2 mmol L^{-1}. Subsequent acetate addition (to ~20.5 mM, day 8) resulted in further consumption of only 0.2 mmol L^{-1}, while current density declined by about 30 % (~ 54 µA cm^{-2}) within 10 h. It is possible that the 8 mL of 1 mol L^{-1} acetate solution, injected through the sample-taking port located in the peripheral recirculation loop upstream of the flow cell, initially passed the biofilm in a relatively undiluted phase leading to an acidic shock and a temporary substrate inhibition. This is consistent with earlier reports of optimal performance at ~14 mmol L^{-1} acetate and a ~15 % performance decrease at 20 mmol L^{-1} (Sapireddy et al., 2021). On day 9, the potential was lowered to 0 mV vs. SHE to test a more energy-efficient operating condition which was not yet reported for *D. acetexigens*. Current density continued to decline but initially with the same slope, so that a direct effect of the potential decrease could not be seen which suggests that the anode remained an effective electron acceptor. OCT analysis (Figure S2b) revealed that the percentage of the anode surface covered

with biofilm (substratum coverage, SC) reached 100 % after 5.2 days and mean biofilm volume increased to 45 µm^3 µm^{-2} (± 17), The maximum current density was measured at a biofilm volume of 25 µm^3 µm^{-2} (± 4) Notably, shortly after full substratum coverage was reached, the current density began to decline. From substrate consumption and current, a coulombic efficiency (CE) of ~96 % was calculated as described in Section 2.4 (integration boundaries: t_0: repolarization of the anode (positive current; day 1.0 after inoculation), t_{end}: last acetate sampling before an increase of acetate concentration (day 7.2 after inoculation), sampling frequency: 1 – 2 day intervals (see Picture S2); Δc= 2.3 mmol L^{-1} acetate, V_R = 0.78 L; $\int_{t_0}^{t} I = 1331.7\, C$), indicating that ~ 96 % of the electrons theoretically gained from the oxidized acetate were transferred to the anode and ~ 4 % were directed to biomass or extracellular polymeric substances. In *G. sulfurreducens*, Moscoviz et al. (2017) likewise reported that only ~ 10 % of electron equivalents from acetate oxidation contribute to biomass formation, which is explained by the relatively low ATP yield ($Y_{ATP/acetate}$ = 2), that drives enhanced catabolic oxidation of acetate to CO_2. (Moscoviz et al., 2017). This is a strong indication that acetate was the only source of electrons and that the hydrogen produced cathodically was not also oxidized, confirming the finding of several authors (Guo et al., 2021; Joshi et al., 2021).

Validation of OCT visualization routines

To ensure that OCT imaging at three predefined positions (A, B, C; 8 × 6 mm^2 each, see Figure 2 I in the main text) adequately represents the biofilm morphology across the entire anode surface (20 × 100 mm, W × L), extended validation experiments were performed.

Hackbarth et al. (2020) demonstrated that OCT datasets acquired at three predefined positions (A, B, C) provide a representative depiction of biofilm morphology across the entire anode. Since their study was conducted under different conditions (cathodic operation with *Kyrpidia spormannii* at 60 °C and 5 bar), it was necessary to verify this approach for anodic biofilms of *D. acetexigens* and *G. sulfurreducens*. Since the applied routine covered only ~7 – 14 % of the anode surface, additional validation was performed in experiment Des2A (see Figure 3 in the main text). On days 6, 8, and 11, OCT datasets spanning the full anode length (100 × 8 mm^2, ~40 % of the surface; Figure 1c III in the main text) were acquired by combining 11 Individual scans (8 × 10 mm^2, W x L). The corresponding height profiles presented in Figure S4 revealed maximal biofilm thicknesses of up to 150 µm, and the biovolumes of all individual scans were calculated and plotted below the profiles.

To confirm that the three imaging positions A, B, and C are sufficient to provide quantitative and representative information on biofilm development across the full anode, the mean biovolume of these positions ($\overline{BV}_{ABC}$) was compared to the mean biovolume ($\overline{BV}_{total}$) of the entire anode length (100 mm). This comparison is shown in Figure S2.

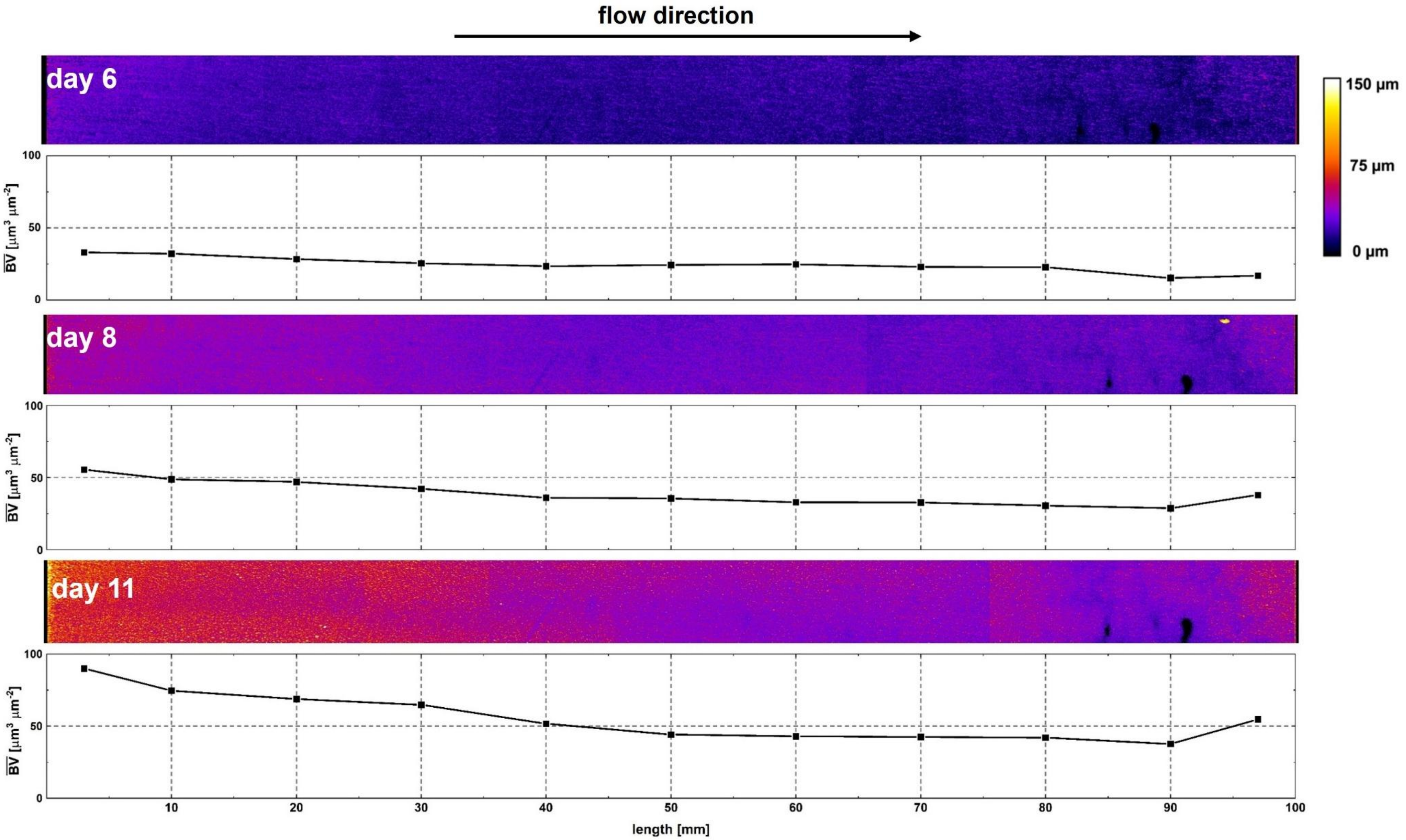

Figure S3: Composite height profiles generated from 11 adjacent 3D OCT datasets covering the full anode length at days 6, 8, and 11 of the *D. acetexigens* cultivation Des2A. The corresponding calculated biovolumes are also shown. For evaluation of the representativeness of the visualized areas used in this study see Figure S4.

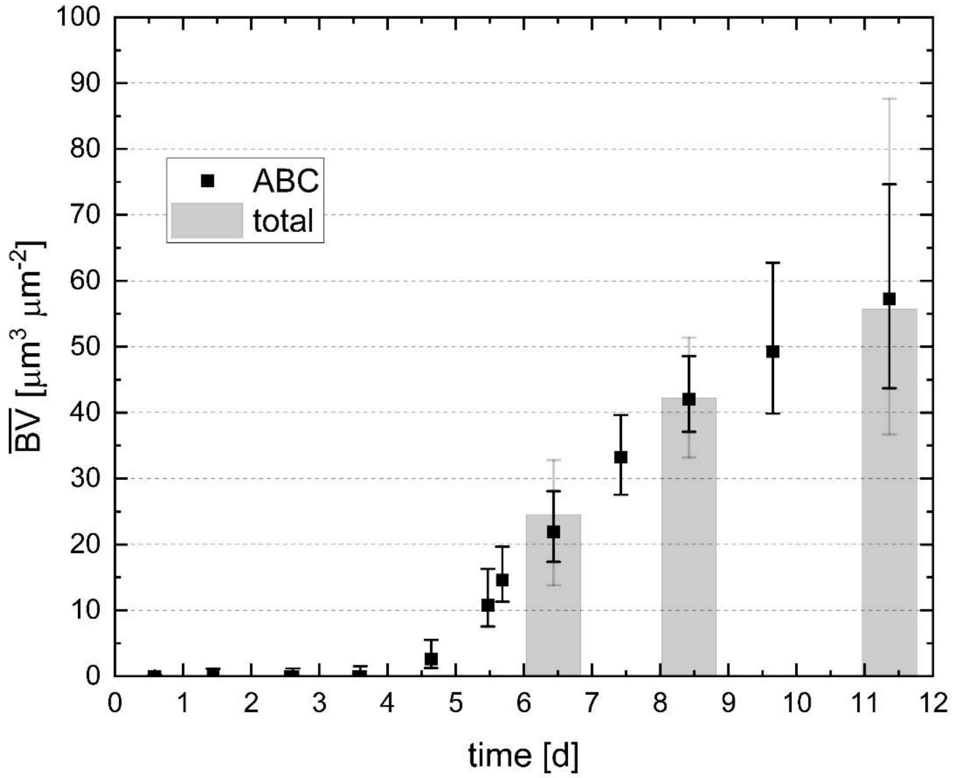

Figure S4: Comparison of the mean biovolume, visualized at positions A, B, and C (black squares) with the mean biovolume across the full anode length (gray bars) for cultivation Des2A. Error bars indicate the respective minimum and maximum values.

The averaged biovolumes at positions A–C (black squares) were compared with the mean volume determined from OCT scans covering the full anode length (100 mm, grey bars), with minimum and maximum values indicated as error bars (Figure S4). On day 6, $\overline{BV}_{total}$was 24.5 μm^3 μm^{-2}, compared to 21.9 μm^3 μm^{-2} at positions A–C, corresponding to a deviation of 10.6 %. This difference can be explained by edge effects near the inlet and outlet of the flow cell, where increased turbulence likely enhanced substrate supply and stimulated local biofilm growth. At later time points (days 8 and 12), deviations of only 0.4 - 2.5 %, demonstrated that the A - C (8 x 6 mm^2, W x L) routine provides a sufficiently accurate representation of biovolume across the anode length, even under varying growth conditions.

To further assess the suitability of the imaging routine, the lateral biofilm morphology was evaluated by comparing standard areas visualized with OCT (8 × 6 mm^2) with fields areas of double width (16 × 6 mm^2) (Figure 1c II in the main text). This analysis was performed for the long-term experiment Des3B (38 days), as shown in Figure S5, to test whether the smaller imaging areas underestimated lateral heterogeneity or provided representative data.

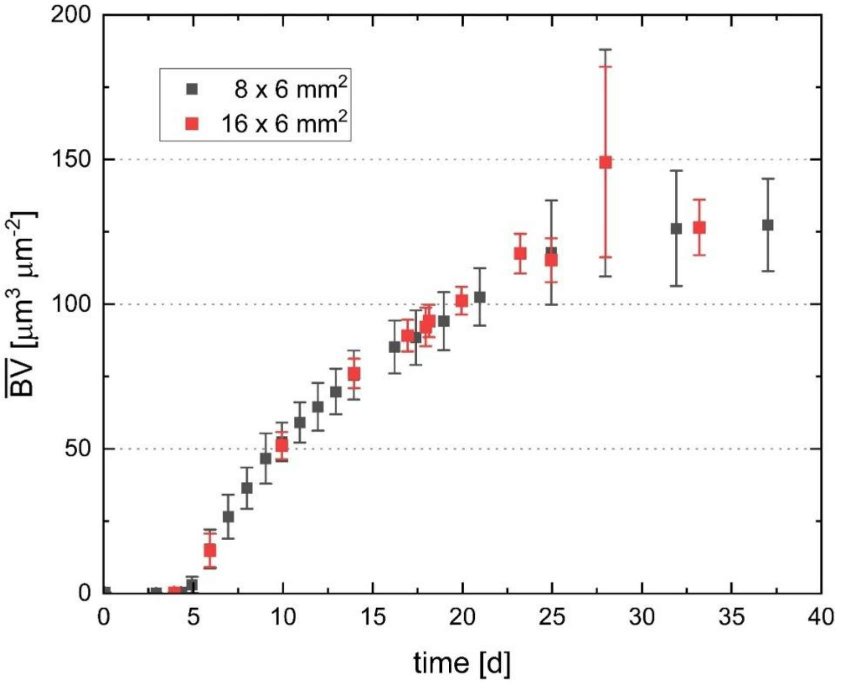

Figure S5: Comparison of the mean biovolume of *D. acetexigens* at positions A, B, and C with an imaging width of 8 mm (gray) and with an imaging width of 16 mm (red). The results shown refer to experiment Des3B.

The mean deviation between the two area sizes was less than 1 % (0.92 %), indicating negligible differences.

Together with the length validation (Figure S4), these results confirm that the A - C imaging routine with visualization areas of 8 × 6 mm^2 (W x L) is representative of the entire biofilm on the 20 × 100 mm^2 anode. Accordingly, all OCT-derived calculations and figures presented in this work can be regarded as robust and reliable.

Taxonomic classification of Des3B and Geo1B at classes level

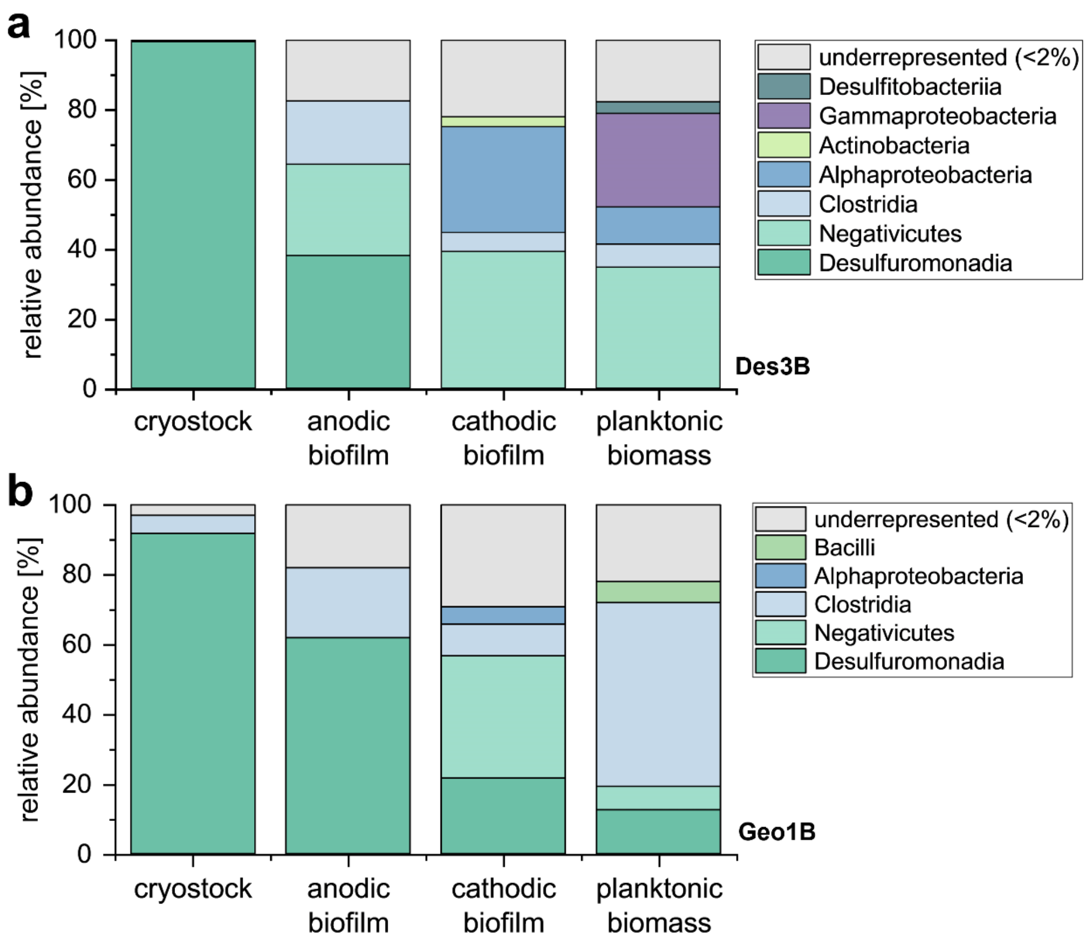

Figure S6: Taxonomic classification of the 16S amplicon sequencing of *D. acetexigens* cultivations Des3B and *G. sulfurreducens* cultivation Geo1B at classes level. Samples were taken from the cryostocks used for inoculation, the anodic and cathodic biofilms, and the planktonic cell fraction of the microbial flow cells. A representation of the taxonomically classified bacterial species is provided in Figure 5 (main text). **a:** *Desulfuromonas acetexigens* cultivation Des3B. **b**: *Geobacter sulfurreducens* cultivation Geo1A.

EtOH positive control (Des3B)

On day 32 after inoculation, when ethanol concentrations had nearly vanished (1.6 mmol L^{-1} at day 26; see Table S2), 1 mL of 70 % ethanol was added to the flow cell. This resulted in an initial ethanol concentration of 10.8 mM directly after injection. As shown in Figure S7, ethanol concentrations subsequently decreased to 5.1 mM by day 37, while acetate concentrations increased by 4.6 mM during the same period.

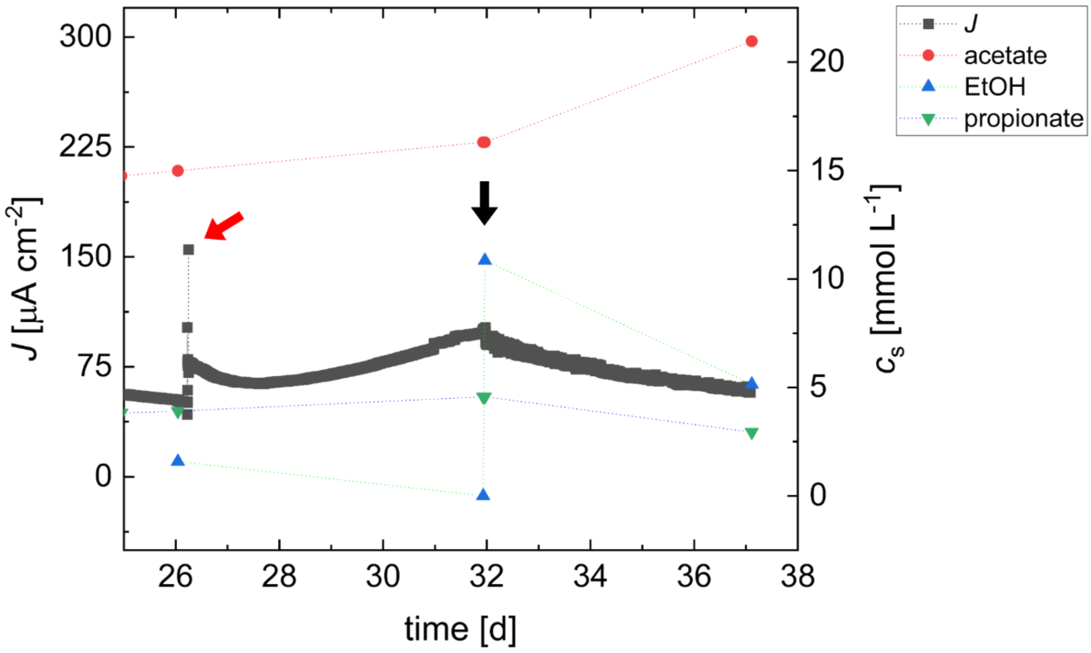

Figure S7: Experiment to verify ethanol fermentation in cultivation Des3B. A pH adjustment from 6.6 to 7.3 was performed on day 26 by adding 2 mL of 0.1 M NaOH (indicated by the red arrow), while targeted addition of 1 mL 70 % ethanol was carried out on day 32 (indicated by the black arrow).

Taking into account the acetate conversion calculated from the current density (0.83 mM), a total acetate production of ~5.4 mM can be assumed. This nearly stoichiometric relationship between ethanol consumption (-5.7 mM) and acetate formation (+5.4 mM) provides strong evidence for ethanol fermentation to acetate under the tested conditions. In addition, Figure S7 shows a decline in propionate concentrations (–1.5 mM) following ethanol addition. The responsible metabolic

pathway and the specific members of the microbial community catalyzing this conversion remain unclear. It cannot be determined whether this effect is directly linked to ethanol fermentation or represents an independent metabolic process of co-occurring bacteria. Nevertheless, the increase of acetate in combination with ethanol depletion supports the interpretation of ethanol fermentation in this system.

EtOH negative control Des4

To examine whether acetate concentrations also increase in the absence of ethanol, the bioelectrochemical cultivation Des4 was performed as a negative control for the electrode mediated ethanol fermentation observed in Des3B. During flow cell setup, medium preparation, and inoculation, particular care was taken to avoid ethanol entry into the system. All sterile steps, including sampling, were conducted under a Bunsen burner flame. *D. acetexigens* was cultivated at an anode potential of 0 mV vs. SHE with an inoculation density of OD_{600}= 0.12. Figure S8 shows the results of this experiment.

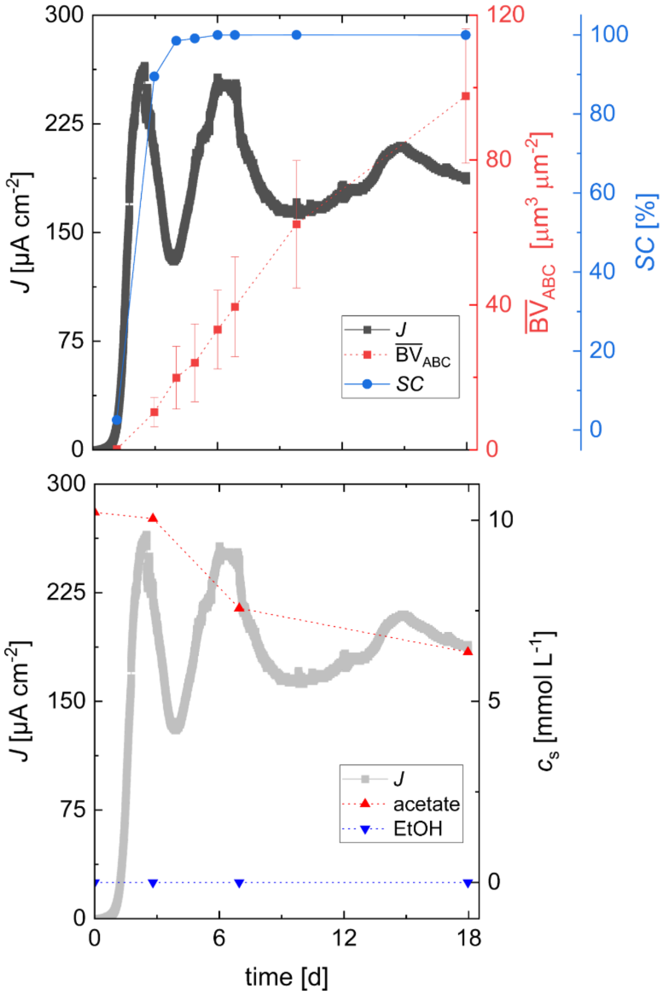

Figure S8: Cultivation of *D. acetexigens* (Des4): negative control without EtOH presence.

The upper graph presents current density, mean biovolume $\overline{BV}_{ABC}$, and substratum coverage (SC) over the 18-day cultivation, while the lower graph depicts acetate and ethanol concentrations. Since the morphological development of Des4 biofilms mirrored observations already described above, it is not further discussed here. As expected, no ethanol accumulation was detected, while acetate concentrations decreased steadily from 10.2 mmol L^{-1} to 6.3 mmol L^{-1} over the cultivation period. Based on current density, the net acetate oxidation was calculated to be 7.2 mmol L^{-1}, which would correspond to a coulombic efficiency (CE) of 2.06 (Section 2.4; integration boundaries: t_0 = repolarization of the anode (positive current, day 1.1 after inoculation), t_{end} last acetate sampling (day 18 after inoculation), sampling frequency: 3 - 10 day intervals (visible in Figure S8); Δc = 3.9 mmol L^{-1} acetate,V_R = 0,84 L; $\int_{t_0}^{t} I = 5216.4\ C$). This CE is unusually high compared to Des1 (~96 %) and previously reported monocultures of *D. acetexigens* in MECs (CE = 98 % ± 2 %; Sapireddy et al., 2021). This discrepancy suggests that Des4 may not have been a monoculture. Unlike Des1, which was directly inoculated from a cryostock (Figure 5 in the main text), Des4 was inoculated from the liquid culture used for Des3B. Consequently, contamination similar to that in Des3B can be assumed. Sequencing data revealed the presence of the homoacetogen *Sporomusa sphaeroides*, which makes it very highly likely that acetogenesis from CO_2 fixation using cathodically produced hydrogen occurred in Des4. Under the condition that no CO_2 was present in the medium at the start of the cultivation, the observed balance corresponds to a conversion ratio of ~1:2. From the measured data, acetate concentrations decreased from $C_{Ac,\,t0}$ = 10.2 to $C_{Ac,\,tE}$ = 6.3 mmol $L^{-1}$by

$\Delta C_{Ac} = 3.9$ mmol L^{-1}, while current-derived calculations suggested a hypothetical oxidation of $\Delta C_{Ac,cons} = 7.2$ mmol L^{-1}acetate. This discrepancy ($C_{VFA,\,prod} = 3.3$ mmol L^{-1}) points to partial acetate regeneration, likely through homoacetogenic activity of the contaminant *Sporomusa* sp., accounting for ~45 % of the consumed CO_2.

Peak-trough-peak discussion

Analysis of Des4 (Figure S8) revealed a characteristic peak–trough-peak pattern in current density, similar to Des1, Des2A, Des3A, Des3B and Des5b (Figure S2; Figure 5 in the main text). Such fluctuations can be explained by rapid metabolic shifts between catabolic and anabolic states (Stockar, 2010; Korth et al., 2020). Even small differences in cultivation parameters (e.g., temperature, gas composition, substrate concentration, inoculum density, or biofilm morphology such as thickness, volume, substratum coverage, roughness, or porosity) may cause cells to exit the initial catabolic phase, where maximum electron release increases current density, and redirect energy toward biomass formation (Korth and Harnisch, 2019). The observed delay of > 1.5 days between $\bar{J}_{max}$and the maximum biofilm accumulation rate (Figure 4 in the main text). shows that, despite their metabolic coupling, current density is not a direct temporal indicator of biomass production. Since current density directly reflects catabolic performance (Stockar, 2010), shifts in electron acceptor availability (Bonanni et al., 2012), biofilm thickness, or full substratum coverage (Hackbarth et al., 2020) may lead to temporary energy storage within the biofilm. Once conditions change, stored energy can be redirected into respiration, resulting in renewed current production (Bonanni et al., 2012; Deng et al., 2018).

These findings highlight that current density and biofilm morphology in MECs are strongly linked to the metabolic state of the biofilm, which is shaped by external factors (Stockar, 2010) and the biofilm morphology itself. Future quantitative expression studies (qPCR) targeting anabolic and catabolic genes at different biofilm growth stages could clarify how cultivation parameters influence

metabolism of EAM communities and thus help optimize reactor conditions for maximal current output (Wagner et al., 2010; Levar et al., 2014; Zacharoff et al., 2016).

Suppl. references:

Bonanni, P.S., Schrott, G.D., Robuschi, L., Busalmen, J.P., 2012. Charge accumulation and electron transfer kinetics in Geobacter sulfurreducens biofilms. Energy Environ. Sci. 5, 6188. https://doi.org/10.1039/c2ee02672d

Deng, X., Dohmae, N., Nealson, K.H., Hashimoto, K., Okamoto, A., 2018. Multi-heme cytochromes provide a pathway for survival in energy-limited environments. Sci. Adv. 4, eaao5682. https://doi.org/10.1126/sciadv.aao5682

Guo, Y., Aoyagi, T., Hori, T., 2021. Comparative insights into genome signatures of ferric iron oxide- and anode-stimulated Desulfuromonas spp. strains. BMC Genomics 22, 475. https://doi.org/10.1186/s12864-021-07809-6

Hackbarth, M., Gescher, J., Horn, H., Reiner, J.E., 2023. A scalable, rotating disc bioelectrochemical reactor (RDBER) suitable for the cultivation of both cathodic and anodic biofilms. Bioresour. Technol. Rep. 21, 101357. https://doi.org/10.1016/j.biteb.2023.101357

Hackbarth, M., Jung, T., Reiner, J.E., Gescher, J., Horn, H., Hille-Reichel, A., Wagner, M., 2020. Monitoring and quantification of bioelectrochemical Kyrpidia spormannii biofilm development in a novel flow cell setup. Chem. Eng. J. 390, 124604. https://doi.org/10.1016/j.cej.2020.124604

Joshi, S.J., Deshmukh, A., Sarma, H. (Eds.), 2021. Biotechnology for Sustainable Environment. Springer Singapore, Singapore. https://doi.org/10.1007/978-981-16-1955-7

Katuri, K.P., Albertsen, M., Saikaly, P.E., 2017. Draft Genome Sequence of Desulfuromonas acetexigens Strain 2873, a Novel Anode-Respiring Bacterium. Genome Announc. 5, e01522-16. https://doi.org/10.1128/genomeA.01522-16

Knoll, M.T., Jürgensen, N., Weiler, J.R., Gescher, J., 2023. Predictability and robustness of anode biofilm to changing potential in microbial electrolysis system. Bioresour. Technol. Rep. 24, 101640. https://doi.org/10.1016/j.biteb.2023.101640

Korth, B., Harnisch, F., 2019. Spotlight on the Energy Harvest of Electroactive Microorganisms: The Impact of the Applied Anode Potential. Front. Microbiol. 10, 1352. https://doi.org/10.3389/fmicb.2019.01352

Korth, B., Kretzschmar, J., Bartz, M., Kuchenbuch, A., Harnisch, F., 2020. Determining incremental coulombic efficiency and physiological parameters of early stage Geobacter spp. enrichment biofilms. PLOS ONE 15, e0234077. https://doi.org/10.1371/journal.pone.0234077

Levar, C.E., Chan, C.H., Mehta-Kolte, M.G., Bond, D.R., 2014. An Inner Membrane Cytochrome Required Only for Reduction of High Redox Potential Extracellular Electron Acceptors. mBio 5, e02034-14. https://doi.org/10.1128/mBio.02034-14

Moscoviz, R., De Fouchécour, F., Santa-Catalina, G., Bernet, N., Trably, E., 2017. Cooperative growth of Geobacter sulfurreducens and Clostridium pasteurianum with subsequent metabolic shift in glycerol fermentation. Sci. Rep. 7, 44334. https://doi.org/10.1038/srep44334

Sapireddy, V., Katuri, K.P., Muhammad, A., Saikaly, P.E., 2021a. Competition of two highly specialized and efficient acetoclastic electroactive bacteria for acetate in biofilm anode of microbial electrolysis cell. Npj Biofilms Microbiomes 7, 47. https://doi.org/10.1038/s41522-021-00218-3

Von Stockar, U., 2010. Biothermodynamics of live cells: a tool for biotechnology and biochemical engineering. J. Non-Equilib. Thermodyn. 35. https://doi.org/10.1515/jnetdy.2010.024

Wagner, R.C., Call, D.F., Logan, B.E., 2010. Optimal Set Anode Potentials Vary in Bioelectrochemical Systems. Environ. Sci. Technol. 44, 6036–6041. https://doi.org/10.1021/es101013e

Zacharoff, L., Chan, C.H., Bond, D.R., 2016. Reduction of low potential electron acceptors requires the CbcL inner membrane cytochrome of Geobacter sulfurreducens. Bioelectrochemistry 107, 7–13. https://doi.org/10.1016/j.bioelechem.2015.08.003
